# Disproportionately High Contributions of 60 Year Old Weapons-^137^Cs Explain the Persistence of Radioactive Contamination in Bavarian Wild Boars

**DOI:** 10.1021/acs.est.3c03565

**Published:** 2023-08-30

**Authors:** Felix Stäger, Dorian Zok, Anna-Katharina Schiller, Bin Feng, Georg Steinhauser

**Affiliations:** † Institute of Radioecology and Radiation Protection, Leibniz Universität Hannover, 30419 Hannover, Germany; ‡ Institute of Inorganic Chemistry, Leibniz Universität Hannover, 30167 Hannover, Germany; § TU Wien, Institute of Applied Synthetic Chemistry & TRIGA Center Atominstitut, 1060 Vienna, Austria

**Keywords:** cesium isotopes, environmental radioactivity, wild boar, nuclear forensics, contaminant persistence

## Abstract

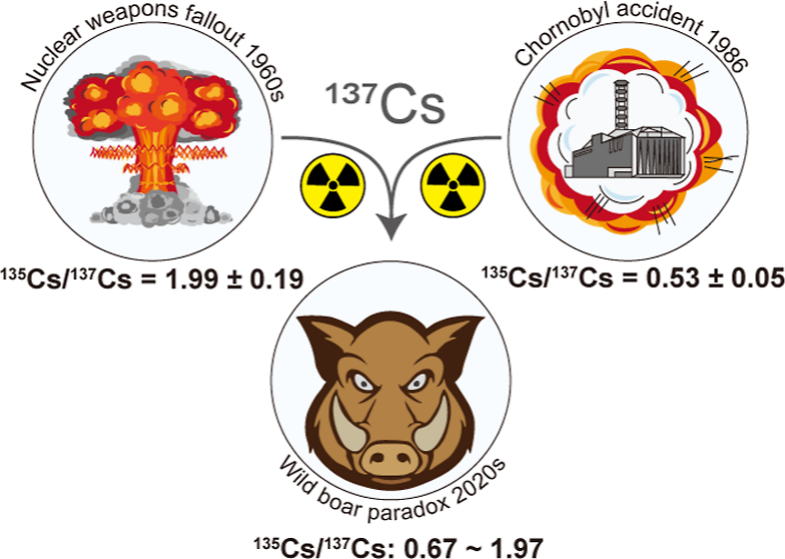

Radionuclides released from nuclear accidents or explosions pose long-term threats to ecosystem health. A prominent example is wild boar contamination in central Europe, which is notorious for its persistently high ^137^Cs levels. However, without reliable source identification, the origin of this decades old problem has been uncertain. Here, we target radiocesium contamination in wild boars from Bavaria. Our samples (2019–2021) range from 370 to 15,000 Bq·kg^–1^^137^Cs, thus exceeding the regulatory limits (600 Bq·kg^–1^) by a factor of up to 25. Using an emerging nuclear forensic fingerprint, ^135^Cs/^137^Cs, we distinguished various radiocesium source legacies in their source composition. All samples exhibit signatures of mixing of Chornobyl and nuclear weapons fallout, with ^135^Cs/^137^Cs ratios ranging from 0.67 to 1.97. Although Chornobyl has been widely believed to be the prime source of ^137^Cs in wild boars, we find that “old” ^137^Cs from weapons fallout significantly contributes to the total level (10–68%) in those specimens that exceeded the regulatory limit. In some cases, weapons-^137^Cs alone can lead to exceedances of the regulatory limit, especially in samples with a relatively low total ^137^Cs level. Our findings demonstrate that the superposition of older and newer legacies of ^137^Cs can vastly surpass the impact of any singular yet dominant source and thus highlight the critical role of historical releases of ^137^Cs in current environmental pollution challenges.

## Abstract 2

Sixty years old ^137^Cs from nuclear weapons fallout contributes significantly to the notorious contamination levels in wild boars in Central Europe that were previously believed to be dominated by Chornobyl.

## Introduction

In the face of climate change, nuclear energy is experiencing a renaissance as a low-carbon option to feed humanity’s hunger for energy.^1^ However, the release of radionuclides into the environment from nuclear accidents or nuclear weapons fallout poses potential threats to public health and societies and economic activities as some radionuclides are capable of persistently contaminating the food chain, resulting in widespread and long-term risk of radiation exposure.^2,3^ The fission product cesium-137 (^137^Cs, half-life *T*_1/2_ = 30.08 y) is a prominent example of such contaminants as it is ubiquitously present in the environment. It originates from the fallout of atmospheric nuclear explosions from the mid-20th century (weapons-^137^Cs) and nuclear accidents, most prominently the Chornobyl^4^ and Fukushima^5,6^ nuclear accidents (reactor-^137^Cs). For safety regulations, many countries have employed strict regulatory limits for ^137^Cs levels in general food products (e.g., EU < 600 Bq·kg^–1^ and Japan: <100 Bq·kg^–1^).^7^ However, although routine radiation surveillance provides essential quantitative information on ^137^Cs contamination levels, the attribution of a contamination to its origins remains poorly understood as the ubiquitous weapons-^137^Cs cannot be distinguished from any reactor-^137^Cs. This analytical challenge impedes the comprehensive understanding of the origin of environmental ^137^Cs contamination, which is a critical prerequisite for a quantitative assessment of the responsibilities for certain ^137^Cs legacies and the establishment of more targeted strategies for environmental remediation and protection. More than ever, with threats of nuclear strikes or accidental releases in the course of the Russo-Ukrainian war, it is now imperative to be able to identify the source of any release of ^137^Cs and evaluate their environmental consequences.

While isotopic signatures of actinides (e.g., uranium and plutonium) have been used successfully to distinguish the contributions between various sources,^8,9^ radiocesium isotopic fingerprints have not yet been applied routinely for source identification. Cesium-135 is an ideal and long-lived candidate (*T*_1/2_ = 2.3 My) after a release, better suited than fast-fading ^134^Cs (*T*_1/2_ = 2.07 y). Also, the production mechanism of ^135^Cs provides more detailed information on the nuclear origin of a contamination, which hence allows attribution of a radiocesium contamination to its source via its distinct ^135^Cs/^137^Cs ratio. Its mother nuclide (^135^Xe) has a large cross-section for thermal neutron capture, resulting in suppressed onset of ^135^Cs under the high neutron flux density of a reactor core.^10^ By contrast, despite the intense but short neutron flux at the moment of a nuclear explosion, ^135^Xe mostly “survives” the explosion because most primary fission products of the 135 isobar are ^135^Te and ^135^I, which have yet to decay to ^135^Xe.^11^ A nuclear explosion hence yields a relatively high ^135^Cs/^137^Cs ratio, whereas a reactor yields a low ratio. Nowadays, analytical protocols for commercial triple quadrupole inductively coupled plasma mass spectrometry (ICP-QQQ-MS) as well as thermal ionization mass spectrometry (TIMS) are available for the precise determination of ^135^Cs/^137^Cs, thus allowing the application of the ^135^Cs/^137^Cs ratio as an isotopic fingerprint in nuclear forensics and environmental tracing studies.^12−19^ In any case, the application of ^135^Cs/^137^Cs as a forensic fingerprint is still far from routine as it requires meticulous chemical separation and sophisticated analytical procedures.

Bavaria, southeastern Germany, is notorious for its heavy ^137^Cs contamination following the Chornobyl nuclear accident.^20^ It was reported that ^137^Cs inventory in surface soil ranged from 10^2^ to 10^5^ Bq·m^–2^ in April 1986 [data from the Federal Office for Radiation Protection (BfS), Germany]. As a potent accumulator of ^137^Cs,^21,22^ regional wild boars (*Sus scrofa*) were subsequently contaminated, and the ^137^Cs activity concentrations in their meat exceeded the regulatory limit by approximately 1–2 orders of magnitude. However, unlike most forest species, which initially also exhibited high ^137^Cs contamination in their bodies followed by a decline with time (i.e., a short ecological half-life),^23,24^^137^Cs levels in wild boars have not shown a significant decline trend since 1986.^20,25^ In certain locations and instances, the decline in contamination levels is even slower than the physical half-life of ^137^Cs.^26^ This phenomenon has been termed “wild boar paradox” and is generally attributed to the ingestion of ^137^Cs accumulating hypogeous fungi (e.g., deer truffle, *Elaphomyces*) by wild boars.^27,28^ Depending on the soil composition, especially clay mineral content,^29^ these underground mushrooms are a critical repository of the downward migrating ^137^Cs. They are one major food item for wild boars, particularly during winter when food on the surface is scarce.^30^ However, due to the lack of convincing evidence for identifying the sources of ^137^Cs, the origins of the persistent contamination in wild boars remains unclear.

Here, we analyzed the ^137^Cs activities together with ^135^Cs/^137^Cs ratios in wild boar meat samples, collected from 11 Bavarian districts during 2019–2021. Reporting the largest environmental sample set of ^135^Cs/^137^Cs to date (*n* = 48), we undertook a critical comparison with the published values and validated the feasibility of utilizing ^135^Cs/^137^Cs for source identification. Using a mixing model, we estimated the contribution of weapons-^137^Cs and reactor-^137^Cs, which not only deepens our understanding of the “wild boar paradox” but may also allow a future location-specific prediction of the evolution of the ^137^Cs contamination in wild boars with time. Lastly, our method can be applied for the traceability of ^137^Cs in any environmental samples in the future.

## Materials and Methods

### Study Regions

**Figure 1:**
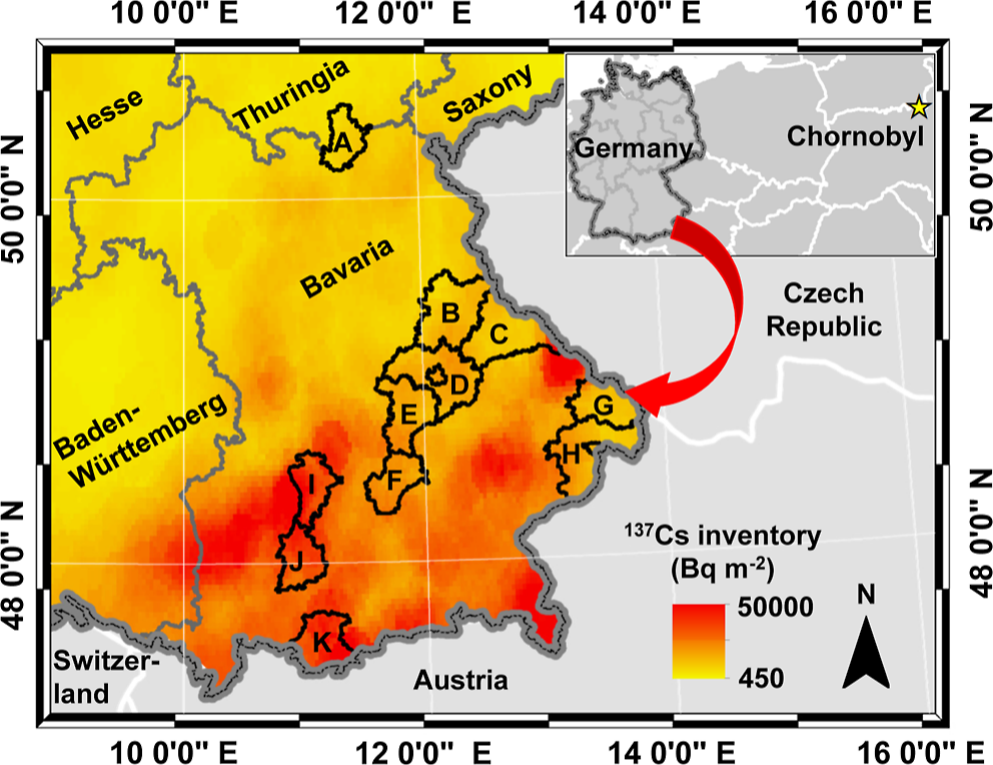
Study regions (districts) in Bavaria. (A) Kronach; (B) Schwandorf; (C) Cham; (D) Regensburg; (E) Kelheim; (F) Freising; (G) Freyung-Grafenau; (H) Passau; (I) Aichach; (J) Landsberg; and (K) Garmisch-Partenkirchen. The ^137^Cs inventory information (Bq·m^–2^) has been derived from the Federal Office for Radiation Protection (BfS), in which the ^137^Cs deposition is corrected to 1986.

Samples of wild boar meat were collected from forested regions of 11 Bavarian districts in southern Germany (Figure 1). At the end of 1984, global ^137^Cs fallout due to atmospheric nuclear explosions led to local ^137^Cs deposition of about 10^3^ Bq·m^–2^.^31^ However, in 1986, considerable additional ^137^Cs fallout from the Chornobyl nuclear accident was deposited on the ground in Bavaria after a long-distance atmospheric dispersion, although the study area is located approximately 1300 km away from the accident site. The input of Chornobyl-^137^Cs immediately increased regional ^137^Cs inventories to 0.5–50 kBq·m^–2^ (reference year: 1986; resolution: 8 × 8 km; source: BfS). Additionally, the local alpine, pedological, and climatic characteristics created a favorable condition for slow ^137^Cs downward migration in the terrestrial environments. For instance, the topsoil classification map created by the Federal Institute for Geosciences and Natural Resources (BGR) showed that clay silt, sandy clay, loamy sand, and loam are the major topsoil textures in the investigated regions.^32^ It is well-known that Cs^+^ ions are strongly bound to the soil’s clay and fine silt fraction, thus preventing rapid vertical migration.^33^ In addition, the historical data recorded by Germany’s national meteorological stations showed that the annual average local rainfall generally varied from about 630 to 1211 mm (median: 776 mm) in the period from 2006 to 2017.^34^ In comparison to the rest of Germany, Southern Bavaria experiences relatively high precipitation rates (>1000 mm) due to the proximity to the Alps. This also caused increased wet deposition (washout of particles) after Chornobyl as well as during global fallout, resulting in a gradual increase of the radiocesium inventory from north to south.

### Sample Collection

Wild boars are traditional game animals in Bavaria. The meat of wild boars (Figure S1 in part 1 of the Supporting Information) was sampled by local Bavarian hunters between fall 2019 and spring 2020, as well as in early 2021. At each site, the fresh muscle samples from targeted wild boar were separated, frozen, and transported to Leibniz University, Hannover, after hunting. Most hunters submitted tongue tissue as an easily available type of tissue with high contamination levels.^35^ In our laboratory, the wild boar muscle samples were thawed at room temperature and then cut into smaller pieces (diameter < 2 cm), while ensuring complete removal of any foreign matrix. In our analytical protocol, briefly, the samples were first dried at 110 °C for 24 h and then heated to 420 °C for 36 h in an oven for final ashing. After necessary cooling, the ashed samples were separately transferred to sealed containers for further storage.

### Reference Materials and Reagents

Two IAEA reference materials, IAEA-330 (spinach) and IAEA-372 (grass), originating from Polessko, Kyiv, Ukraine, were used for verifying the measured ^135^Cs/^137^Cs ratio in our laboratory. The “Mixed nuclide solution 7601” from Eckert & Ziegler Nuclitec GmbH was applied for calibrating the counting efficiency of the specific geometry in γ-ray spectrometry for ^137^Cs determination. Merck Millipore Milli-Q water (18.2 MΩ·cm) and guaranteed grade regents, including HNO_3_ (69%, Carl Roth), HCl (37%, Carl Roth), and NH_3_ (20%, Carl Roth), were used to prepare solutions, which were then utilized in sample digestion and radiochemical analysis. Ammonium molybdophosphate powder [AMP, H_12_Mo_12_N_3_O_40_P·*x* H_2_O (*x* ≈ 3), ACS >95%] purchased from Alfa Aesar was used for the Cs extraction. To remove the potentially interfering elements from the ashed samples, Dowex 1-X8 (100–200 mesh) anion exchange resin purchased from Alfa Aesar and the cation exchange resin AG 50 W-X8 (100–200 mesh) from BioRad Laboratories, Inc. were prepared for sample purification.

### ^137^Cs Activity Measurement

The ashed sample was homogenized and filled into a plastic container for γ-ray measurement. The sample’s ^137^Cs activity was measured by a high-purity germanium (HPGe) gamma detector, using the 661.7 keV γ-peak. Gamma ray efficiency calibration was performed using Eckert & Ziegler’s certified “Mixed nuclide solution 7601.” The detector has a counting volume of 131 cm^3^ with a relative detection efficiency of 28% and a resolution of 1.9 keV at the 1332 keV ^60^Co γ-ray peak. The software Genie 2000 was used for evaluating the γ-ray spectra of each sample. Based on the calibration files prepared in our laboratory, the measured data were corrected with the counting geometry and energy. Gamma-ray self-attenuation was corrected by using the “top-down method” (see part 2 of the Supporting Information). Besides, a physical decay correction was also performed for all measured data to the sampling day.

### Analysis of the ^135^Cs/^137^Cs Ratio in Ashed Samples

The analysis protocol established by Zok et al.^11^ was used in this section, which mainly encompasses three steps: (I) cesium extraction; (II) cesium purification; and (III) ^135^Cs/^137^Cs ratio determination by ICP-QQQ-MS. The detailed process is described in part 3 of the Supporting Information (Table S1). In part 4 of the Supporting Information, the amounts of reference samples (IAEA-330 and IAEA-372) used for the analysis are listed (Table S2), and the cross-comparison of ^135^Cs/^137^Cs ratios in reference materials with published values^11,17,36−39^ (QA/QC) is described (Tables S3 and S4 and Figure S2).

### Statistical Analysis

“Median ± standard deviation” is used for the description of the data distribution. Spearman correlation analysis and linear regression analysis were used to quantitatively study the relationship between variables and ^137^Cs data. One-way analysis of variance (ANOVA) was applied to evaluate the difference between significant differences in ^137^Cs activity concentration and ^135^Cs/^137^Cs among wild boar characteristics groups. All the statistical analysis was implemented in SPSS v.22.

## Results and Discussion

### ^137^Cs Contamination in Bavarian Wild Boar

**Figure 2:**
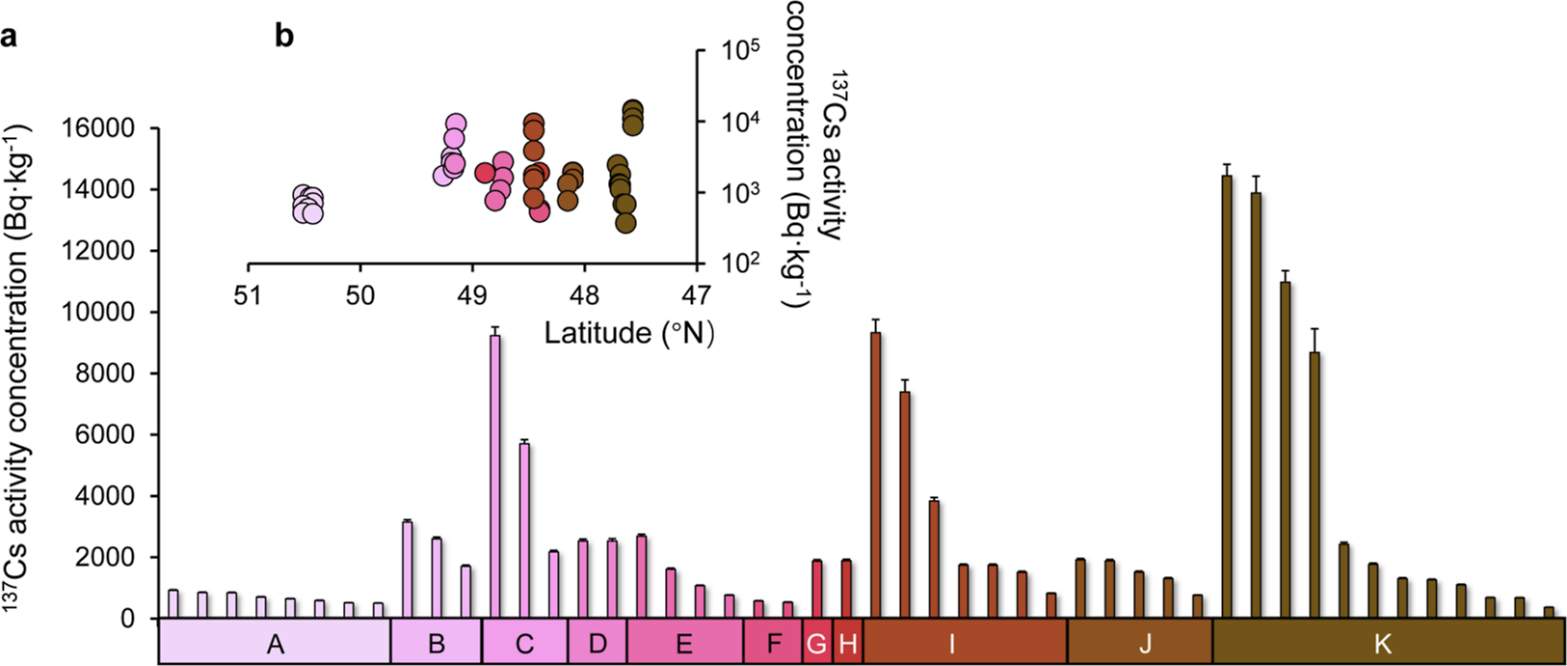
^137^Cs contamination in wild boars. (a) Fresh weight ^137^Cs activity concentrations (Bq·kg^–1^) in wild boar meat collected from 11 districts in Bavaria; all measured ^137^Cs corrected to the sampling day; the error bar reflects the uncertainty by gamma spectrometry (*k* = 1). (b) Spatial distribution between the measured ^137^Cs activity concentrations (Bq·kg^–1^) and their corresponding latitude (°N). Study regions: (A) Kronach; (B) Schwandorf; (C) Cham; (D) Regensburg; (E) Kelheim; (F) Freising; (G) Freyung-Grafenau; (H) Passau; (I) Aichach; (J) Landsberg; and (K) Garmisch-Partenkirchen.

The detailed information about sampling and the measured results are summarized in Tables S5 and S6 in part 5 of the Supporting Information. Overall, the fresh weight activity concentration of ^137^Cs in wild boar meat collected from 11 Bavarian districts varied between 0.37 and 14 kBq·kg^–1^ (Figure 2a), with a median of 1.7 kBq kg^–1^ (SD: 3.5 kBq·kg^–1^, *n* = 48), in which about 88% of measured data were above the regulatory limit according to German law and all data exceeded the Japanese limit. In addition, a spatial heterogeneity of ^137^Cs contamination levels was observed between various Bavarian districts, with the range of coefficients of variation (CVs) from 23 to 113% (excluding regions with a sample size < 3). In the Garmisch-Partenkirchen region (region K, southern Bavaria), we observed minimum and maximum ^137^Cs levels in wild boars with the widest range (14 kBq·kg^–1^) and the highest CV (115%, *n* = 12). In northern Bavaria (Kronach, region A), we found a relatively narrow ^137^Cs variability (0.50–0.92 kBq·kg^–1^, *n* = 8) and the lowest ^137^Cs contamination (0.67 ± 0.16 kBq·kg^–1^). By contrast, region C (Cham, eastern Bavaria) contributed the heaviest ^137^Cs levels in this study (5.70 ± 3.53 kBq·kg^–1^, *n* = 3). From a temporal perspective, hardly any significant decline trend can be found in ^137^Cs activity concentrations between our samples (2019–2021) and the historical record of ^137^Cs contamination in wild boar during similar seasons since 2001 (Figures S3 and S4, part 6 of the Supporting Information), which is consistent with the observation of persistent ^137^Cs contamination in wild boars from Austria.^25^ To explore the potential sources responsible for the persistent ^137^Cs contamination in wild boars, we compared measured ^137^Cs with the inventories in the study regions. With negligible contributions from Fukushima,^40^ we considered reactor-^137^Cs from Chornobyl and weapons-^137^Cs as the major sources in Bavaria. For weapons-^137^Cs, the cessation of atmospheric tests resulted in no noteworthy new weapons-^137^Cs fallout after the last test in 1980. Nowadays, the baseline of airborne ^137^Cs (<10 μBq·m^–3^ in Germany) contributes insignificantly to the relatively high inventory in soil^40,41^ and consequently in wild boars. Considering that the above-mentioned contamination level is orders of magnitude greater than the ^137^Cs inventories reported in less contaminated regions,^41,42^ it suggests a substantial impact of Chornobyl-^137^Cs on the Bavarian ecosystem. This is consistent with reports of Chornobyl-^137^Cs dominating the total ^137^Cs inventory in Austria by 90%.^43^ Besides, we found notable geographical differences in ^137^Cs inventories among investigated districts with a declining trend from south to north (*r* = −0.93, *P* < 0.01). Moreover, these inhomogeneous ^137^Cs spatial patterns are also discovered in any specific district with a range of CVs from 12% (Freising, region E) to 63% (Cham, region C). However, a simple regression analysis showed no latitudinal pattern for ^137^Cs levels in wild boars and no obvious correlation between the ^137^Cs activity concentrations and topsoil inventories (Figure 2b). This phenomenon was also reported in ^137^Cs contaminations in American honey^44^ as well as Japanese wild boars.^45^ We therefore suggest that due to various factors, such as animal mobility, activity inventory in soil, soil type,^20,46^ land use (e.g., agricultural soil vs forest soil),^6,47^ animal access to agricultural areas with lower ^137^Cs levels in crops than in wild plants or mushrooms growing in forests,^20^ heterogeneity of deposition, season of sampling,^30^ etc.,^48^ no simple model can correlate topsoil ^137^Cs inventories and the resulting activity concentration in the animal tissue.

### ^135^Cs/^137^Cs Ratio Profile and ^137^Cs Source Identification

**Figure 3:**
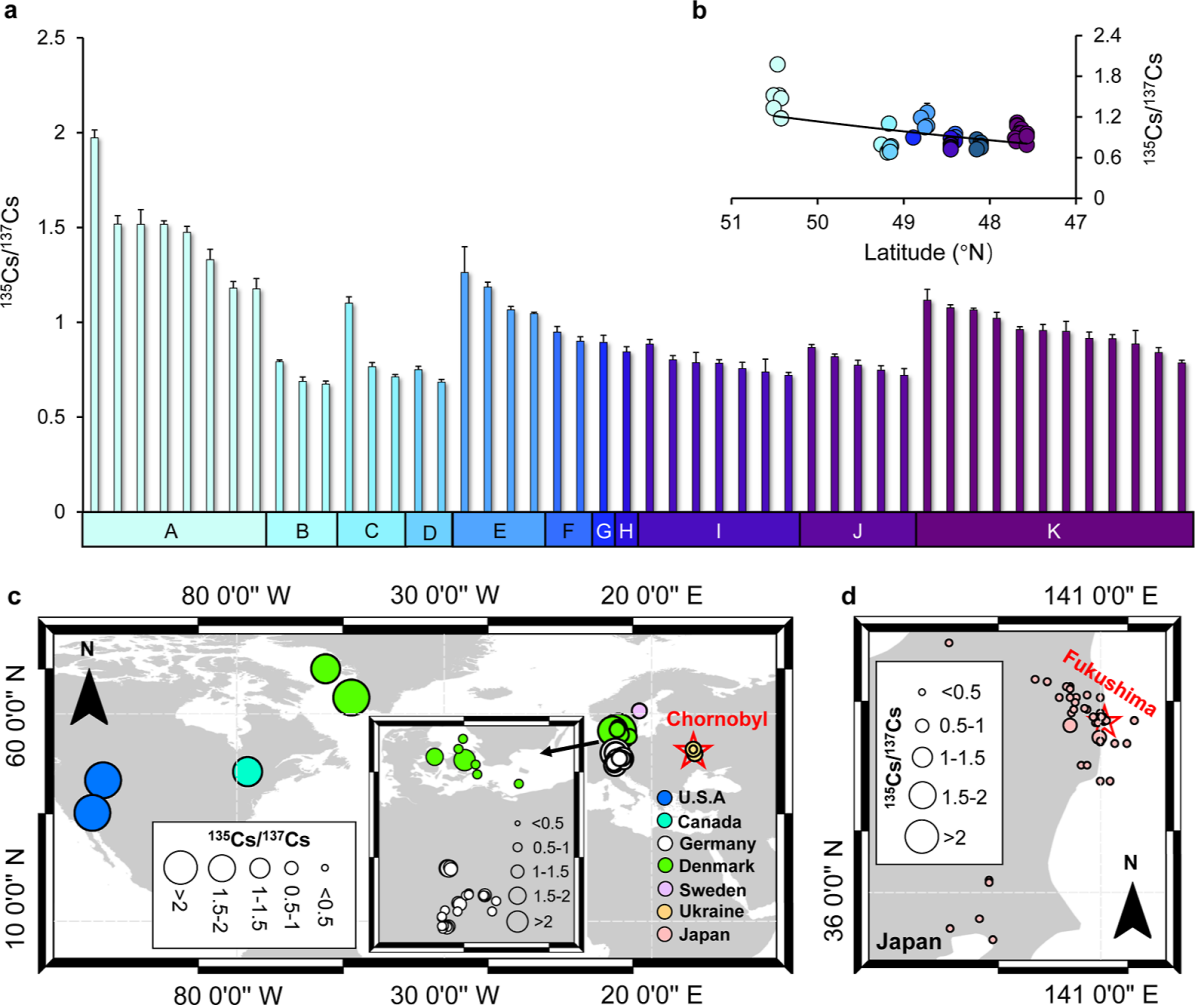
Distribution of ^135^Cs/^137^Cs ratios in the environment. (a) General profiles of ^135^Cs/^137^Cs ratios in wild boar meat collected from Bavaria, the error bar is the measured uncertainty given by ICP-QQQ-MS (*k* = 1). (b) Spatial distribution between the measured ^135^Cs/^137^Cs ratio and their corresponding latitude (°N). Comparison of ^135^Cs/^137^Cs ratios in environmental samples reported in Europe and North America (c), as well as Japan (d). The red stars in the map represent the two regions that experienced serious nuclear accidents (i.e., Chornobyl, Ukraine and Fukushima, Japan). All the data were decay-corrected to March 11, 2011. Study regions: (A) Kronach; (B) Schwandorf; (C) Cham; (D) Regensburg; (E) Kelheim; (F) Freising; (G) Freyung-Grafenau; (H) Passau; (I) Aichach; (J) Landsberg; and (K) Garmisch-Partenkirchen.

For a convenient comparison, all ratios were corrected to March 11, 2011, which is the date of the last major radiocesium source affecting the environment (Fukushima nuclear accident). We propose this date as the reference for decay correction in ^135^Cs/^137^Cs studies. The measured ^135^Cs/^137^Cs ratios ranged from 0.67 to 1.97 (Figure 3a and Table S5, 0.90 ± 0.28), with the highest ratio found in the northern region (Kronach, region A) and the lowest ratio from the central part of Bavaria (Schwandorf, region B). Similarly, like the radiocesium inventory in Bavaria decreasing from south to north, there is a pronounced geographical pattern in ^135^Cs/^137^Cs ratios, in which the latitude-dependent decline is confirmed (Figure 3b, *R*^2^ = 0.35, *P* < 0.01, *n* = 48), whereas a relatively poor significant correlation between the topsoil ^137^Cs inventories and ^135^Cs/^137^Cs ratios (*R*^2^ = 0.18, *P* < 0.01) is apparent. Unlike the measured ^137^Cs activity concentrations, the ^135^Cs/^137^Cs ratio’s variability is relatively narrow, with the CV varying from 4 to 24% (median: 9%). More interestingly, correlation analysis showed a significant positive relationship between the ^135^Cs/^137^Cs ratio’s CV and topsoil ^137^Cs inventories’ CV in these regions (*R*^2^ = 0.69, *P* < 0.01, *n* = 8), suggesting the dependence of ^135^Cs/^137^Cs ratios’ difference on the non-uniform spatial distribution of topsoil ^137^Cs inventories. Considering that such non-uniform patterns are typically attributed to the deposition of Chornobyl-^137^Cs, we here used the map of Chornobyl-derived ^137^Cs from Meusburger et al.^49^ and found a significant heterogeneity in Chornobyl-derived ^137^Cs in our study regions, with the CV ranging from 48 to 105%. The great variability of the ^135^Cs/^137^Cs ratios in this study is thus thought to reflect the variable contributions of ^137^Cs sources in Bavaria.

To better apply the measured ^135^Cs/^137^Cs ratios for the discrimination of the two radiocesium sources, we systematically compared our values with all reported ^135^Cs/^137^Cs ratios over the last decades (part 7 of the Supporting Information, Tables S7–S9, excluding any IAEA reference materials) and plotted the measured value with their locations, as shown in Figure 3c,d. It can be seen that the ^135^Cs/^137^Cs ratios obtained from countries that experienced major nuclear accidents (i.e., Ukraine and Japan, range: 0.31–0.73, 0.37 ± 0.08, *n* = 72) are much lower with a narrow range than those obtained from regions far away from any locations with releases from nuclear accidents (e.g., USA,^50^ Canada,^51^ and Greenland,^52^ range: 1.21–2.84, 1.89 ± 0.50, *n* = 9). By contrast, the ^135^Cs/^137^Cs ratios in samples from other European countries (e.g., Germany,^11^ Denmark,^52^ and Sweden^52^) were in between the fingerprint signature of weapons-^137^Cs and reactor-^137^Cs (range: 0.54–2.18, 0.95 ± 0.30, *n* = 57), thus indicating mixing. In the case of the present study, there is a significant gap in the range of ^135^Cs/^137^Cs ratios between the minimum value (0.67) and the maximum value (1.97) measured in Bavarian wild boars. This inconsistency may be explained by the uptake of radiocesium from mixed sources.

The ^135^Cs/^137^Cs ratio in different countries may be affected by the weapons’ and test’s characteristics (type, yield, and distance between the sampling location and ground zero).^11,50,53^ For instance, the median ratio in the USA (2.21 ± 0.40, *n* = 3) is about 41% higher than that in Canada (1.57 ± 0.30, *n* = 4). Therefore, we here adopted the ratio obtained from the historical human lung tissue (Vienna)^11,54^ as the ^135^Cs/^137^Cs fingerprint for central Europe, which likely represents the integral signature in a European setting. These samples were collected in the 1960s, so its ^135^Cs/^137^Cs ratio is only governed by weapons fallout (1.99 ± 0.19, *n* = 5). In this scenario, the higher ^135^Cs/^137^Cs ratios observed in Kronach (region A, 1.50 ± 0.25, *n* = 8), compared to that in other regions, and the relatively low ^137^Cs contamination in wild boars together suggest that, here, Chornobyl-^137^Cs was not the dominant source in this region. By contrast, some regions have high ^137^Cs contamination and a relatively low mean ^135^Cs/^137^Cs ratio, such as Cham (region C, ratio range: 0.77 ± 0.21, *n* = 3), which implies that Chornobyl-^137^Cs mostly accounts for the local ^137^Cs contamination. To better display the spatial distribution of ^135^Cs/^137^Cs ratios in the study area, we plotted the measured values on the ^137^Cs deposition map derived from BfS data (Figure S5 in part 8 of the Supporting Information).

### Mixed Legacy ^137^Cs from Global Fallout and Chornobyl Nuclear Accident

**Figure 4:**
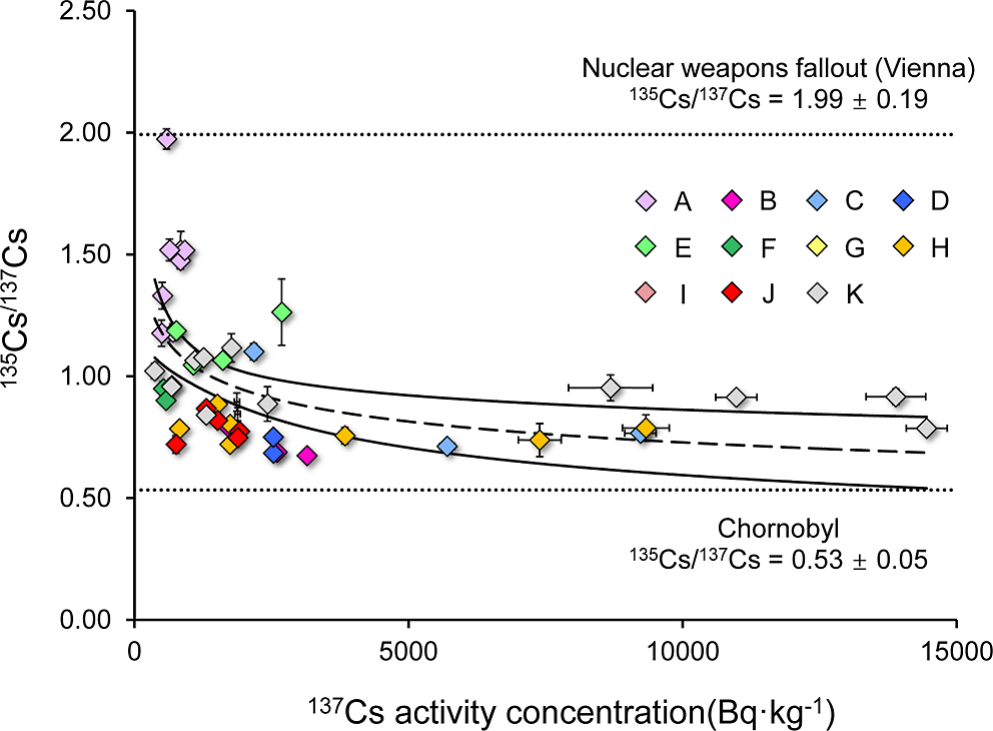
General relationship between the measured ^137^Cs activity concentrations and ^135^Cs/^137^Cs ratios in wild boar meat from Bavaria. The error bars are the measured uncertainty of the ^137^Cs activity concentration and ^135^Cs/^137^Cs ratio. The solid line in is the 95% confidential interval of the fitting curve (dashed line). The dotted lines represent the ^135^Cs/^137^Cs ratio in the signature of weapons fallout (1.99 ± 0.19) and nuclear accident (0.53 ± 0.05).

Considering that the ^135^Cs/^137^Cs ratios observed in wild boars are negatively related to the amount of Chornobyl-^137^Cs ingested from their habitat, we expected that there would be a negative relationship between the ^137^Cs activity concentrations and ^135^Cs/^137^Cs ratios in samples collected from Bavaria. To validate this idea, we applied the regression analysis for two arrays, and as expected, we found a negative correlation (Figure 4, *P* < 0.01). However, the ^135^Cs/^137^Cs ratios estimated by the fitting curve do not match the measured value in most cases, and the *R*^2^ is only about 0.25. Metabolic variabilities may explain why certain specimens of wild boars may accumulate or maintain ^137^Cs levels more efficiently than others, whereas the ^135^Cs/^137^Cs ratio will be unaffected, leading to such a mismatch between the ^137^Cs activity concentration and the ratio. Nevertheless, statistical analysis showed no significant difference in either the ^137^Cs activity concentration or the ^135^Cs/^137^Cs ratio among our wild boars’ characteristics (see Figure S6 in part 9 of the Supporting Information).

It is well-known from Chornobyl and Fukushima that topsoil ^137^Cs is rapidly adsorbed onto (clay) minerals and gradually migrates vertically,^33,55^ until it reaches further potential accumulators such as underground fungi.^27^ The global fallout from atmospheric nuclear explosions peaked in 1964, more than 20 years prior to Chornobyl’s fallout. Due to the time span between both events, the actual ^137^Cs inventory in greater soil depths differs from that in the topsoil across the regions. Moreover, the availability of ^137^Cs in subterranean species (e.g., fungi) may further amplify the differences in the spatial pattern of ^137^Cs as its downward migration rate and accumulation process varies with the local environmental conditions, such as the increased likelihood for precipitation in the alpine regions of Southern Bavaria. Thus, another interpretation for the poor correlation between ^137^Cs activity concentrations and ^135^Cs/^137^Cs ratios is that the regional difference in ^137^Cs availability complicates the correlation of two variables using a single model. In other words, different contribution percentages of two independent ^137^Cs sources may result in identical ^137^Cs contamination levels, thus weakening the explanation of the observed phenomenon using a single regression model. This hypothesis has been tested in Figure S7 (part 10 of the Supporting Information) where significant differences in the relationship between ^137^Cs levels and ^135^Cs/^137^Cs ratios (sample size ≥ 2) were observed in the studied regions.

### Application of the ^135^Cs/^137^Cs Ratio for Source Contribution Estimation

**Figure 5:**
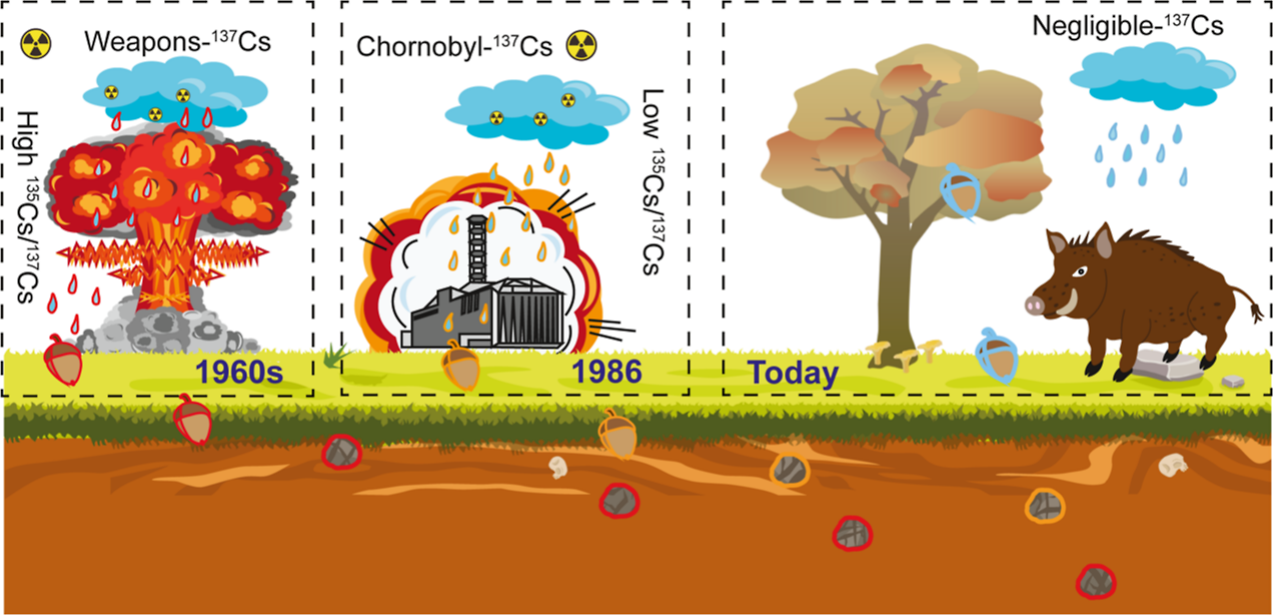
Conceptual mechanism diagram of different ^137^Cs sources mixed and ingested by wild boar. The red, orange, and blue boundaries for items are for the weapons-^137^Cs, Chornobyl-^137^Cs, and ^137^Cs-free sources, respectively. Attribution: one graphic in this diagram was designed by Macrovector—Freepik.com. Two graphics were adapted from the Media Library of University of Maryland Center for Environmental Science. Reprinted or adapted with permission under a Creative Commons CC BY-SA 4.0 license from Tracey Saxby, Integration and Application Network (http://ian.umces.edu/media-library). Copyright 2005 and 2011, respectively.

We propose that two ^137^Cs sources (nuclear weapons fallout and Chornobyl) have mixed in the Bavarian soil, the release maxima of which were about 20–30 years apart. To visualize this mixing process more intuitively, we propose a conceptual mechanism diagram (Figure 5). Up until the mid-1990s, atmospheric nuclear explosions released about 545–765 PBq ^137^Cs into the upper atmosphere,^14^ and by stratosphere–troposphere mass exchange and atmospheric deposition, this weapons-^137^Cs with a high ^135^Cs/^137^Cs ratio gradually reached the surface and entered the food chain. Conversely, 85 PBq ^137^Cs were released by the Chornobyl nuclear accident.^56^ This radiocesium with a low ^135^Cs/^137^Cs ratio dispersed across Europe and deposited especially in Alpine regions, resulting in the mixing of two sources.^57^ While the deposition conditions of the two radiocesium releases depended on local weather and microclimate conditions (e.g., precipitation), downward migration in the pedosphere depends on the biogeochemical system’s complexity (e.g., presence of fungi^58^), resulting in significant regional differences in ^137^Cs migration, mixture, and accumulation. The situation for wild boars is further complicated by their mobility, which allows them to cover areas with higher and lower contamination, possibly with a variable mixing degree. Instead of focusing on food sources (e.g., fungi), it hence appears more practical to focus on the actual, integral contamination in the wild boar itself as it represents the final outcome of an equation with virtually countless factors (season, food sources, regional cesium availability, soil properties, animal mobility, etc.).

**Figure 6:**
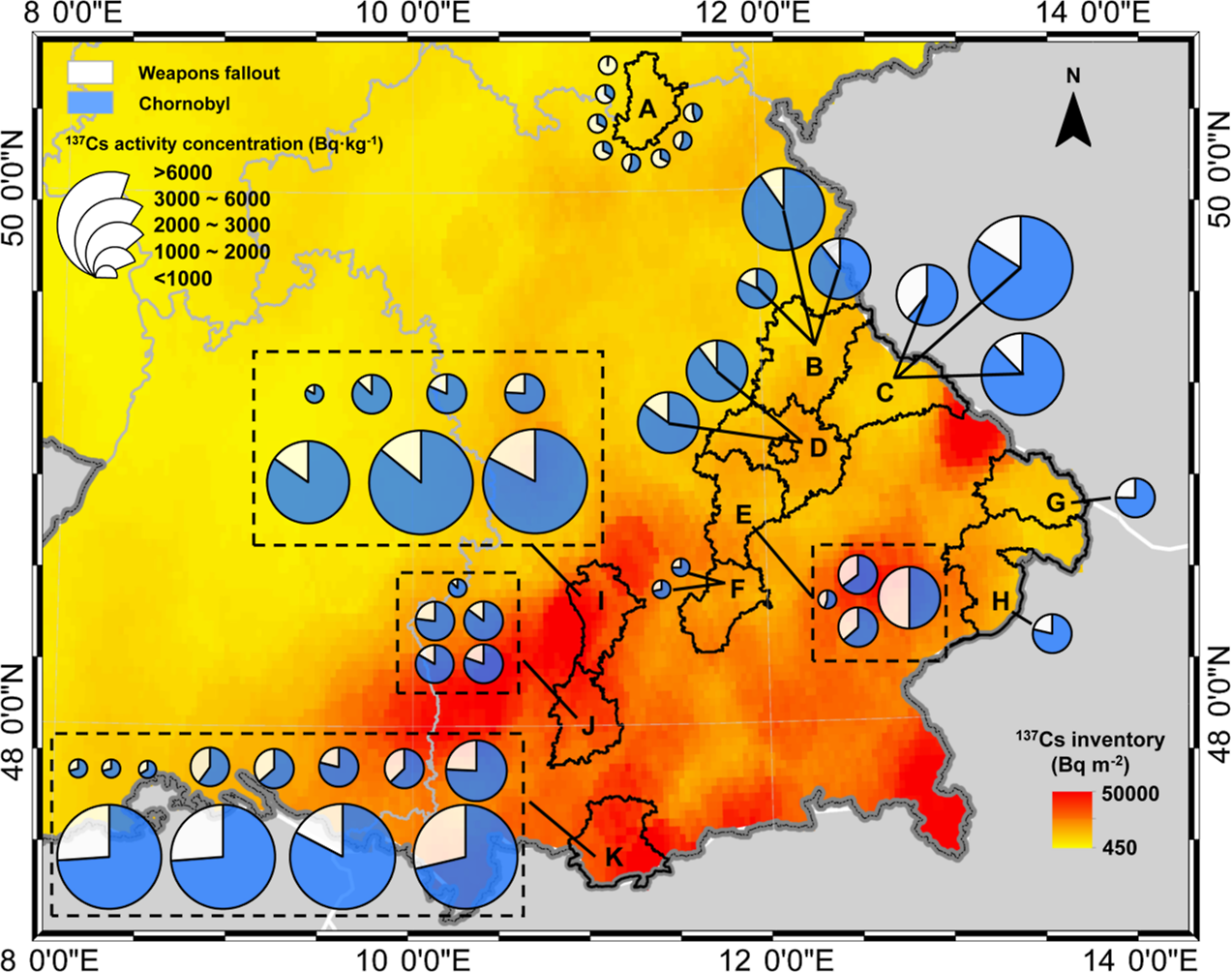
^137^Cs activity concentrations in wild boars and their contributions from weapons fallout and the Chornobyl nuclear accident. The light yellow and blue are the contribution percentages of weapons-^137^Cs and Chornobyl-^137^Cs, respectively. The ^137^Cs inventory information (Bq·m^–2^) has been derived from BfS (^137^Cs deposition is decay-corrected to 1986).

Here, we applied a binary mixing model for evaluating the contributions of weapons-^137^Cs and Chornobyl-^137^Cs in wild boars using the characteristic ^135^Cs/^137^Cs ratios (Table S7). With the signature of fallout (*R*_f_, value of 1.99) and Chornobyl (*R*_c_, value of 0.53), the cesium from fallout (*P*_s_) can be estimated by putting the measured ratio (*R*_s_) into the equation *P*_s_ = (*R*_s_ – *R*_c_)/(*R*_f_ – *R*_c_). Moreover, we plotted the ^137^Cs activity concentrations and radiocesium contributions in wild boars for all samples on the basis of the ^137^Cs deposition map derived from BfS (Figure 6).

The mixing model showed that the median ^137^Cs contributions in boars from weapons fallout and Chornobyl are approximately 25 and 75%, respectively (see Table S10 in part 11 of the Supporting Information). Compared with the Chornobyl-^137^Cs contributions in top soils estimated by plutonium isotopes (ca. 60–90%),^32^ although there is a good linear relationship between the two data sets (part 12 of the Supporting Information, Figure S8, *R*^2^ = 0.63, *P* < 0.01, *n* = 48), the predicted Chornobyl contribution percentages estimated by cesium isotopes are found slightly lower. Particularly in northern Bavaria (region A), the contributions from Chornobyl-^137^Cs are significantly lower than in the rest of Bavaria (range: 1–56%, median: 34%), but the ^137^Cs contamination level in wild boars still exceeds the regulatory limit in 62.5%. Although it is still debated whether or not the persistent ^137^Cs contamination in Bavarian boars spanning decades has its roots in fungal species, we can now present isotopic evidence that the atmospheric weapons fallout that has been residing in our environment for more than 60 years is still affecting radioactive contamination levels in wild boars. More unexpectedly, in Cham (region C), Kelheim (region E), and Garmisch-Partenkirchen (region K), we found that about 40–50% of ^137^Cs contamination in some wild boar samples originated from the weapons fallout. Therefore, our findings provided visual evidence for the interpretation that persistent ^137^Cs contamination in Bavarian wild boars is also related to the six decades old global weapons fallout in our ecosystem.

### Disproportionate Contributions of ^137^Cs from Weapons Fallout

**Figure 7:**
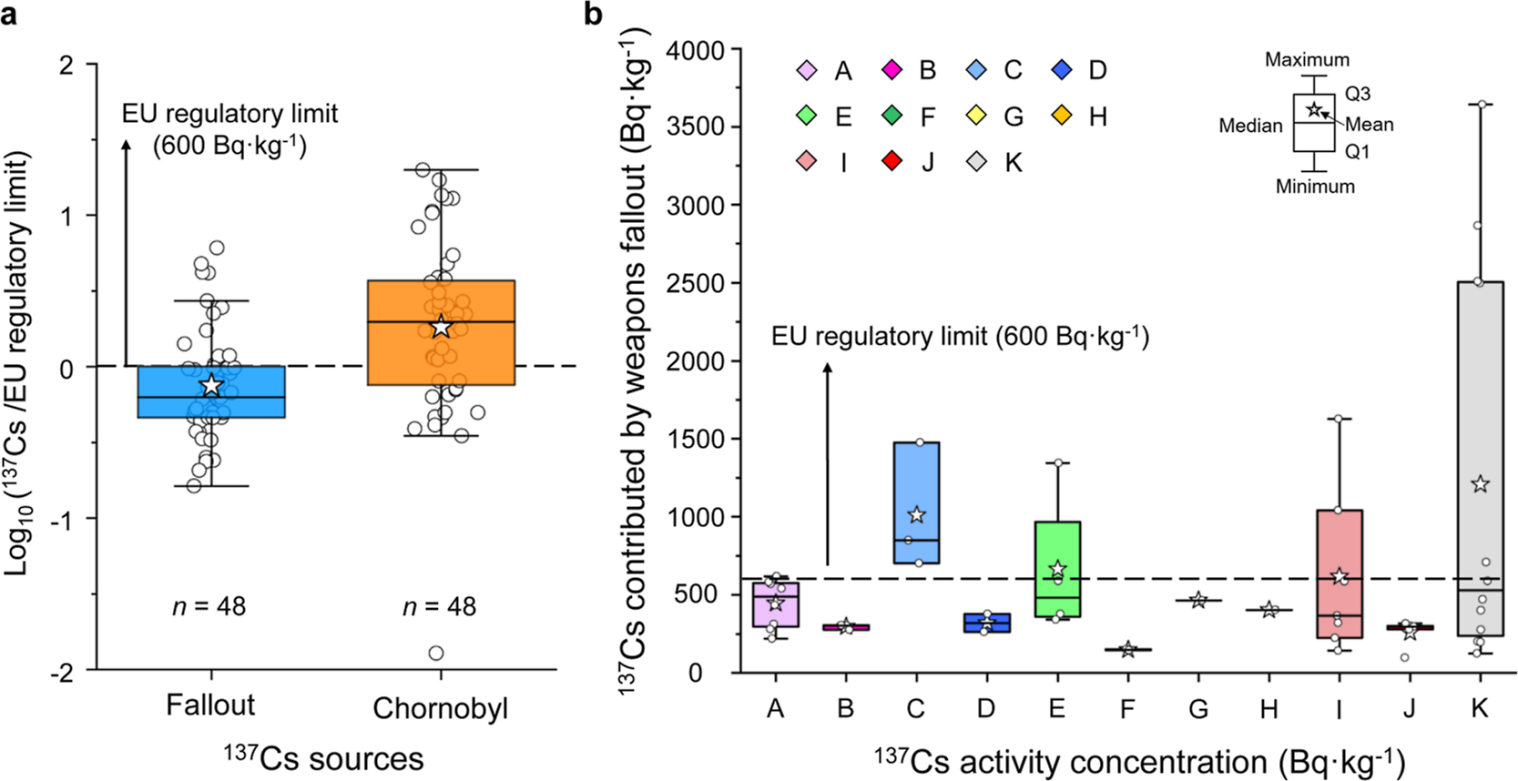
Weapons-^137^Cs activity concentrations in wild boars. (a) General comparison between the weapons-^137^Cs activity concentrations and Chornobyl-^137^Cs activity concentrations (normalized to the EU regulatory limit, 600 Bq·kg^–1^). (b) Weapons-^137^Cs activity concentrations in Bavarian wild boars in various study regions. The white circles are the measured data (*n* = 48). Study regions: (A) Kronach; (B) Schwandorf; (C) Cham; (D) Regensburg; (E) Kelheim; (F) Freising; (G) Freyung-Grafenau; (H) Passau; (I) Aichach; (J) Landsberg; and (K) Garmisch-Partenkirchen.

In order to further evaluate the weapons-^137^Cs contribution to the radioactive contamination in Bavarian wild boars, we calculated the weapons-^137^Cs activity concentration by using the estimated contribution percentage and the total ^137^Cs concentration (Table S10). As shown in Figure 7a, although Chornobyl-^137^Cs remains the overall more significant contributor to wild boar contamination, about 25% of wild boar samples exhibit such significant contributions from weapons-^137^Cs that the fraction of weapons-^137^Cs alone is high enough to exceed the European regulatory limit (600 Bq·kg^–1^). Spatially, these samples originated from regions A (*n* = 1), C (*n* = 3), E (*n* = 1), I (*n* = 2), and K (*n* = 5), respectively (Figure 7b). Further analysis suggests that high weapons-^137^Cs activity concentration occur in certain regions where the total contamination level of boars is very high (>5000 Bq·kg^–1^), with the relative contribution of weapons-^137^Cs between 12% (region C) and 29% (region K). On the other hand, a similar pattern is observed via an alternative pathway in certain regions where the total contamination level is relatively low (<3000 Bq·kg^–1^) but the relative contribution of weapons-^137^Cs is high (e.g., ∼67% in region A or ∼50% in region E). In any case, no assumptions about the contribution of old weapons-^137^Cs should be made just by a given ^137^Cs deposition. For example, both of the above patterns were observed in region K, where the ^137^Cs deposition exceeds 30,000 Bq·m^–2^. In contrast, the contribution of weapons-^137^Cs to the total activity concentration was found to be below 20% in region B with a relatively low ^137^Cs deposition inventory (∼9000 Bq·m^–2^). We suggest that this phenomenon may be caused by a certain randomness in the food selection and uptake of ^137^Cs by wild boars since regional ^137^Cs availability and environmental factors can generate multiple combinations of the two ^137^Cs legacies. Despite great challenges in revealing a detailed picture of the persistently high ^137^Cs contamination in Bavarian wild boars, our findings demonstrate that the six decades old weapons-^137^Cs alone is still capable of yielding significant contamination levels that exceed the regulatory limit in wild boars today. Therefore, for the scientific community of radioecology, the disproportionate weapons-^137^Cs contribution may provide new insights into the use of effective half-lives to describe ^137^Cs behavior in the terrestrial environment as the value is also governed by the various ^137^Cs legacy sources and their mixing process in the region.

## Implications and Perspectives

Our work reveals deeper insights into the notorious radiocesium contamination in Bavarian wild boars beyond the total radionuclide quantification only. Using ^135^Cs/^137^Cs as a direct isotopic fingerprint, we were able to show that the mixed ^137^Cs legacy from Chornobyl and nuclear weapons fallout is responsible for the persistence of high contamination levels. With the effective half-lives of ^137^Cs in wild boars being longer than the physical half-life of ^137^Cs, this phenomenon sometimes must have appeared like a violation of the law of radioactive decay. By implementation of a mixing model, our findings demonstrate that weapons-^137^Cs contributed between 12 and 68% in those samples that exceeded the regulatory limit. The unusually high levels in wild boars not only legitimate rigid regulatory control for human food safety, they are partly (21% of samples) also above the conservative screening benchmark levels (i.e., 10 μGy·h^–1^) for boars themselves.^21^

Although the weapons-^137^Cs has resided in the environment for at least 60 years (i.e., two physical half-lives of ^137^Cs) and its contribution as a pollutant of central Europe has generally been regarded as negligible compared to that of Chornobyl, our work provides the forensic evidence showing that this underestimated ^137^Cs legacy can accumulate in certain environmental media along with more recent reactor-^137^Cs releases. Both contributors form an intense ^137^Cs source that exceeds the contribution from any singular, yet dominant source in the area (like Chornobyl in the case of Bavaria). This mixed source is the main supplier to wild boars in the winter season and in turn the main reason for the persistent ^137^Cs contamination in Bavarian wild boars. After several single-source studies, this is the first time that ^135^Cs/^137^Cs has been used to demonstrate the accumulation of radiocesium legacies from different nuclear sectors in ecosystem species and that the effects of such “superimposed” radioactive contaminations have been caught while they are transmitted through the food chain of biological communities, eventually to human consumers of game meat. The recognition of this deleterious environmental impact thus provides new insights for the radioecological research community as policy makers may need to consider a multitude of ^137^Cs contributors to the total inventory in an ecosystem and take them all into account for holistic risk assessment.

Any future ^137^Cs release from nuclear accidents or nuclear explosions will add to the historical ^137^Cs legacy over time and further aggravate the current contamination situation. According to the International Atomic Energy Agency, 56 nuclear power reactors are currently under construction across the world,^59^ thus underscoring the role of nuclear power in the future global energy portfolio. With the intensifying war between Ukraine and Russia, much concern has been expressed about the terrible consequences of a nuclear war or a combat-triggered nuclear accident. Once released, radiocesium will remain in the environment for generations and impact food safety immediately and, as shown in our study, for decades. Any additional releases will cause further accumulation and mixing with older sources, making it necessary to understand the underlying mechanisms of the biogeochemical cycling of radiocesium. For example, the impact of soil properties on mixing of different radiocesium sources has not yet been understood sufficiently. Consequently, more efforts are still needed to better understand the sources, inventories, environmental fates, and ecological risks of radiocesium.

Having proven a powerful tool in complex radioecological questions, this study highlights the outstanding potential of ^135^Cs/^137^Cs for the distinction of present or future ^137^Cs sources. Possible applications include other environmental ^137^Cs repositories such as mushrooms,^60,61^ honey,^44^ or sediments.^62^ Lastly, this study illustrates that strategic decisions to conduct atmospheric nuclear tests 60–80 years ago still impact remote natural environments, wildlife, and a human food source today. A similar, long-lasting consequence can be expected from Chornobyl-^137^Cs deposited in central Europe that will have a longer impact than the relatively short ecological half-lives of ^137^Cs suggest.

## Supporting Information Available

Photos of wild boars in study regions (part 1); “top-down method” for correcting gamma-ray self-attenuation (part 2); detailed procedure of ^135^Cs/^137^Cs analysis (part 3); cross-comparison of ^135^Cs/^137^Cs ratios in reference materials (part 4); detailed information on sampling and measured data about the ^137^Cs activity concentration, ^137^Cs/^133^Cs ratio, and ^135^Cs/^137^Cs ratio (part 5); historical variation of ^137^Cs activity in Bavarian wild boars (part 6); comparison of measured ^135^Cs/^137^Cs ratios (part 7); spatial distribution of the ^135^Cs/^137^Cs ratio in Bavarian wild boars (part 8); effects of wild boar characteristics on radiocesium dynamics (part 9); relationship between the ^137^Cs activity concentration and ^135^Cs/^137^Cs ratio in each district (part 10); mixing model application (part 11); and comparison of the Chornobyl-^137^Cs contribution with another method (part 12) (PDF)
