# Supplementary material for: Disproportionately High Contributions of 60 Year Old Weapons-137Cs Explain the Persistence of Radioactive Contamination in Bavarian Wild Boars
Source: Environ Sci Technol. 2023 Aug 30;57(36):13601–11. doi: 10.1021/acs.est.3c03565 (PMC10501199; doi:10.1021/acs.est.3c03565)
Supplement: Supplementary file 1 — es3c03565_si_001.pdf [file es3c03565_si_001.pdf]

Disproportionately High Contributions of 60-Year-Old Weapons-^{137}Cs Explain the Persistence of Radioactive Contamination in Bavarian Wild Boars

Felix Stäger[1], Dorian Zok[1], Anna-Katharina Schiller[1], Bin Feng[2,3*], Georg Steinhauser[3*]

1. Institute of Radioecology and Radiation Protection, Leibniz University Hannover, Herrenhäuser Str. 2, 30419 Hannover, Germany
2. Institut für Anorganische Chemie, Leibniz Universität Hannover, Hannover, 30167, Germany
3. TU Wien, Institute of Applied Synthetic Chemistry & TRIGA Center Atominstitut, 1060 Vienna, Austria

[*]Corresponding author: Bin Feng, binfjf@outlook.com; Georg Steinhauser: georg.steinhauser@tuwien.ac.at

This supporting information includes 12 parts, in which 7 text descriptions, 8 supplementary figures, and 10 supplementary tables were attached.

Supporting Information Guide

1. Typical habitat of highly contaminated Bavarian wild boars

- **Figure S1.** Wild boars in forested regions of Bavaria.

2. “Top-down-method” for correcting gamma-ray self-attenuation

- **Text 1.** Method description of “Top-down-method”

3. Procedure of $^{135}Cs/^{137}Cs$ analysis

- **Text 2**. General procedure of $^{135}Cs/^{137}Cs$ analysis
- **Table S1.** Analytical parameter settings in ICP-QQQ-MS.

4. Cross-comparison of $^{135}Cs/^{137}Cs$ ratios in reference materials

- **Text 3.** Cross-comparison of $^{135}Cs/^{137}Cs$ with reported value (QA/QC).
- **Table S2.** The amounts of reference samples (IAEA-330 and IAEA-372) used for the analysis.
- **Table S3.** Comprehensive comparison of $^{135}Cs/^{137}Cs$ ratio for IAEA reference material (IAEA-330).
- **Table S4.** Comprehensive comparison of $^{135}Cs/^{137}Cs$ ratio for IAEA reference material (IAEA-372).
- **Figure S2.** Comprehensive comparison of $^{135}Cs/^{137}Cs$ ratio in IAEA reference materials (IAEA-330 and IAEA-372) with the ratios reported in previous studies.

5. Detailed information of sampling sites and data

- **Table S5**. Detailed information of sampling in the study area
- **Table S6**. Detailed information of samples

6. Historical variation of ^{137}Cs activity in Bavarian wild boars

- **Text 4.** Temporal variations in ^{137}Cs activity concentration in Bavarian wild boars

- **Figure S3**. Seasonal variations in ^{137}Cs activity concentration in wild boars collected from Aichach from 2001 to 2022.
- **Figure S4.** Comparison of annual variations in ^{137}Cs activity concentration in wild boars collected from Bavaria from 2001 to 2022.

7. Comparison of measured $^{135}Cs/^{137}Cs$ ratios with reported values

- **Text 5.** Method description of $^{135}Cs/^{137}Cs$ ratios comparison
- **Table S7.** Reported $^{135}Cs/^{137}Cs$ ratio in the environmental samples collected from Ukraine.
- **Table S8.** Reported $^{135}Cs/^{137}Cs$ ratio in the environmental samples collected from Japan.
- **Table S9**. Reported $^{135}Cs/^{137}Cs$ ratio in the environmental samples collected from regions without nuclear accident.

8. Spatial distribution of $^{135}Cs/^{137}Cs$ ratio in Bavarian wild boars

- **Figure S5. $^{135}Cs/^{137}Cs$ ratio in Bavarian wild boars**

9. Effects of wild boar characteristics on ^{137}Cs activity and $^{135}Cs/^{137}Cs$ ratio

- **Text 6**. Discussion of the influences of wild boar characteristics on radioecesium dynamics.
- **Figure S6.** Effect of individual characteristics (gender, age, and weight) on the ^{137}Cs contamination levels and $^{135}Cs/^{137}Cs$ ratio in Bavarian wild boars.

10. Relationship between ^{137}Cs activity concentration and $^{135}Cs/^{137}Cs$ ratio in each district

- **Figure S7.** Relationship between the measured ^{137}Cs activity concentrations and $^{135}Cs/^{137}Cs$ ratios in Bavarian wild boar in each district.

11. Estimation of ^{137}Cs contribution by mixing model

- **Text 7**. Method description of mixing model

- **Table S10.** Estimation of ^{137}Cs from the nuclear weapon fallout to total ^{137}Cs activity concentration in the meat of Bavarian wild boars.

12. Comparison of Chornobyl-^{137}Cs contribution estimated by two methods

- **Figure S8.** Comparison of Chornobyl-^{137}Cs contribution between our method (^{135}Cs/^{137}Cs) in wild boar and the plutonium method in top soil.

1. Typical habitat of highly contaminated Bavarian wild boars

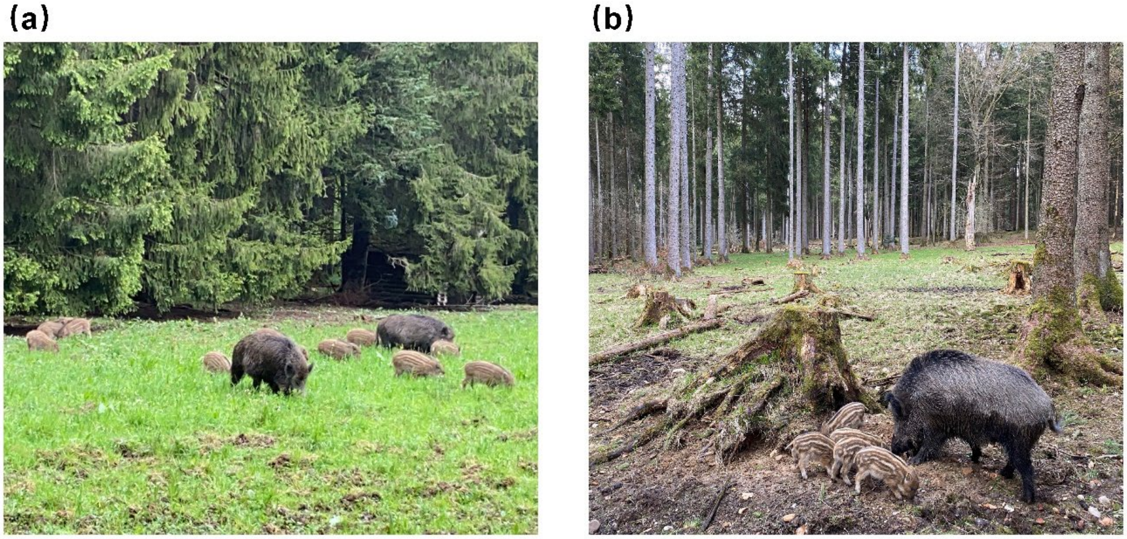

Figure S1. Wild boars in forested regions of Bavaria. (a) A herd of wild boars at the edge of the forest. (b) A mother sow and squeakers foraging in contaminated top soil. © Joachim Reddemann, reprinted with permission from the copyright holder.

2. “Top-down-method” for correcting gamma-ray self-attenuation

A plastic petri dish was completely filled with the ashed sample, sealed with PARAFILM® “M” by Merck and measured for gamma radiation on top of a coaxial HPGe detector. Determination of the detector’s efficiency was performed by measurement a filter paper with the identical diameter as the petri dish was infused with a defined amount of a certified Eckert & Ziegler GmbH multiple gamma-ray-emitting solution (Mixed nuclide solution 7601), which also contains ^{137}Cs. In order to take gamma-ray self-attenuation into account the sample was measured with the soaked filter paper on top and underneath the petri dish. In order to estimate the self-shielding, the efficiencies of the respective top and down measurements were calculated, see Equations (1) and (2), respectively. For the calculation of the total efficiency of the measurement, the logarithmic mean of the two individual efficiencies was used, Equation (3). The self-attenuation-corrected activity of the sample was finally determined via the measured count rate (cps_{sample}), gamma intensity (η), and the total efficiency ($\varepsilon_{\text{total}}$) (4). This method was implemented by J.-W. Vahlbruch and verified with certified IAEA-soil as well as in various round robin tests.

$$cps_{\mathrm{filter,i}} = cps_{\mathrm{sample+filter,i}} - cps_{\mathrm{sample}} \quad (1)$$

$$\varepsilon = \frac{cps_{\mathrm{filter,i}}}{A_{\mathrm{filter}}} \quad (2)$$

$$\varepsilon_{\mathrm{total}} = \frac{\varepsilon_{\mathrm{down}} - \varepsilon_{\mathrm{top}}}{\ln\left(\frac{\varepsilon_{\mathrm{down}}}{\varepsilon_{\mathrm{top}}}\right)} \quad (3)$$

$$A_{\mathrm{sample}} = \frac{cps_{\mathrm{sample}}}{\varepsilon_{\mathrm{total}} \cdot \eta} \quad (4)$$

3. Procedure of $^{135}Cs/^{137}Cs$ analysis

The general procedure of $^{135}Cs/^{137}Cs$ analysis used in this study was described in our previous work,[1] which mainly includes three steps: (I) cesium extraction; (II) cesium purification; (III) $^{135}Cs/^{137}Cs$ ratio determination by ICP-QQQ-MS.

(I) Cesium extraction. The ashed samples were first transferred into 110 mL PTFE vessels with about 10 mL HNO_3 (69%) for microwave digestion. In the MARS 6 system (CEM Corporation), the vessels were heated from room temperature up to 160 °C for 20 min, and held for 30 min. Afterwards, the aqueous solution was filtered through a filter paper (Whatman, pore size < 2 µm) and collected in a polyfluoroalkoxy (PFA) beaker. To minimize the cesium loss during sample transfer, about 15 mL Milli-Q water was added into the digestion vessels to rinse any residual sample solution. The filtrated and rinsing solution were combined and the mixture was evaporated to dryness and re-dissolved in 10 mL HNO_3 (1.6 M). For the quantification of the recovery yield of cesium by this analytical protocol, the mass of the resulting solution was recorded and about 50 µL aliquot were taken from the mixture. This tracer was diluted to 5 mL by 2% HNO_3 for further ICP-MS determination. Finally, the volume of mother solution was increased to 45 mL using 1.6 M HNO_3 and 35 mg of AMP was added for Cs extraction. After about 60 min stirring, a cellulose acetate syringe filter with pore

size of 1.2 μm was employed in separating the AMP(Cs) and residual solution. The AMP(Cs) in the filter was then re-dissolved by 10 mL of NH_3 (1.5 M) for further purification.

(II) Cesium purification. Commercially available anion and cation ion exchange resins were used for removing the interfering elements (e.g., Sn, Sb, Mo, and Ba) from the re-dissolved samples. Prior to the experiment, about 3.5 mL anion exchange resin (DOWEX 1X8) was first pre-cleaned with about 20 mL NH_3 (1.5 M). The re-dissolved AMP(Cs) solution was then loaded onto the prepared anion resin column. Since the oxo-anions of Sn, Sb, and Mo can be trapped by this resin, this process allowed cations, such as Cs^+ and Ba^{2+}, to elute to the loading effluent. Additional 10 mL NH_3 (1.5 M) was added to the column for washing out the residual Cs. The combined solutions were evaporated to dryness and re-dissolved in a PFA baker loading 10 mL NH_3 (0.15 M) for second separation.

The final separation of Cs and Ba was performed by a cation exchange resin (AG 50WX8). Similarly, prior the use, about 3.5 mL of cation exchange resin were pre-cleaned in resin column by 4 M HNO_3 first. After a necessary rinse with Milli-Q water, the cleaned resin was conditioned with 25 mL NH_3 (0.15 M), following the second re-dissolved solution packed in the column. About 10 mL NH_3 (0.15 M), 10 mL Milli-Q water, and 15 mL HCl (1.5 M) were successively used in the

resin washing. In this case, Ba^{2+} would be retained on the resin and Cs+ would be allowed to elute. Ultimately, the eluate was evaporated again and re-dissolved in 2 mL 2 % HNO_3 for $^{135}Cs/^{137}Cs$ ratio determination. The solution was weighed again and about 50 µL tracer was taken and diluted for the determination of the recovery yield, which followed the procedure developed by our lab previously.[1]

(III) $^{135}Cs/^{137}Cs$ ratio determination by ICP-QQQ-MS. All measurements were performed using the 8900 ICP-QQQ-MS (Agilent Technologies, Inc.) with MassHunter 4.4 software and an SPS4 auto-sampler. Solutions of blank samples, standard samples and environmental samples were all prepared using Milli-Q water and sub-boiled HNO_3. For the measurement of the $^{135}Cs/^{137}Cs$ ratio, a mixture of reaction gas, including He (5N, Linde GmbH) and N_2O (2N, Messer Group GmbH) was used under the triple quadrupole mode, whereas for the quantification of the recovery yield, a single quadrupole mode without reaction gas was adopted. Detailed measurement parameters for the concentration measurement and isotope ratio determination are listed in Table S1. Following Zok et al.,[1] the plasma's mass bias was corrected to a 5 $\mu g \cdot L^{-1}$ Eu solution (1000 $mg \cdot L^{-1}$, Alfa Aesar) and the measured $^{135}Cs/^{137}Cs$ ratios were corrected to March 11, 2011 for better comparison with previously published values.

Table S1. Analytical parameter settings in ICP-QQQ-MS for the determination of concentration and isotope ratio.

Item	Parameter	Concentration	Isotopic ratio
Sample Introduction	Stabilization time	40 s	15 s
	Sample uptake	45 s, 0.3 rpm	30 s, 0.5 rpm
	Probe rinse	90 s, 0.3 rpm	90 s, 0.3 rpm
Spectrum Acquisition	Q2 peak pattern	3 Points	3 Points
	Replicates	5	5
	Sweeps/Replicates	100	1000
Plasma	RF power	Low Matrix	1550 W
	RF matching		1.4 V
	Sample depth		10.0 mm
	Nebulizer gas		1.07 $L \cdot min^{-1}$
	Nebulizer pump		0.1 rps
	S/C temperature		2 °C
	Makeup gas		0.25 $L \cdot min^{-1}$
Lenses	Extract 1	-12.8 V	-23 V
	Extract 2	-250 V	-250 V
	Omega bias	-140 V	-145 V
	Omega lens	9.4 V	8.6 V
	Q1 exit	3.0 V	1.0 V
	Cell focus	-3.0 V	1.0 V
	Deflect	15.2 V	6.8 V
	Cell entrance		-62 V
	Cell exit		-51 V
	Plate bias		-52 V
Q1	Q1 bias	-9.0 V	-2 V
	Q1 pre-filter	-9.5 V	-5.6 V
	Q1 post-filter	-10.0 V	-10 V
Cell	He flow		1 $L \cdot min^{-1}$
	3rd gas flow (N_2O)		6% (0.6 $L \cdot min^{-1}$)

REFERENCE

1. Zok, D, et al. Determination of characteristic vs anomalous $^{135}Cs/^{137}Cs$ isotopic ratios in radioactively contaminated environmental samples. *Environ. Sci. Technol.* **2021**, 55, 4984-4991.

4. Cross-comparison of $^{135}Cs/^{137}Cs$ ratios in reference materials

Because there is no available standard sample for $^{135}Cs/^{137}Cs$ determination, the cross-comparison of the reference materials (IAEA-330 and IAEA-372) was conducted between our measurement and published values. The amounts of reference samples used for the analysis was listed in Table S2. The reference samples measured in this work and the comparison with reported values (10, 49-53) were listed in Table S3 (IAEA-330) and Table S4 (IAEA-372). Figure S2 shows the visualized comparison results. In general, the $^{135}Cs/^{137}Cs$ ratios in two reference materials measured in our laboratory are in good agreement with the available reported values.

Table S2. The amounts of reference samples (IAEA-330 and IAEA-372) used for the analysis.

IAEA reference material	No.	Sample amounts (g) [a]
IAEA-372	1	1.31009
	2	1.31962
	3	1.20059
	4	1.37436
	5	1.24416
	6	1.25105
	7	1.26591
	8	1.21788
IAEA-330	1	5.37310
	2	5.03999
	3	5.04449
	4	5.29204
	5	5.34687

a. Weighing resolution of balance: 0.00001 g

Table S3. Comprehensive comparison of $^{135}Cs/^{137}Cs$ ratio for IAEA reference material (IAEA-330) [a]

IAEA reference material	$^{135}Cs/^{137}Cs$		Statistics				Instrument	Ref.
	Ratio	Uncertainty	Min	Median	SD	Max		
IAEA-330	0.57	0.06	0.49	0.51	0.03	0.57	ICP-QQQ-MS	This work
	0.49	0.05						
	0.51	0.05						
	0.57	0.06						
	0.51	0.11						
	0.53	0.02	0.52	0.52	0.00	0.53	TIMS	1
	0.52	0.02						
	0.52	0.02						
	0.49	0.01	0.47	0.48	0.01	0.49	ICP-QQQ-MS	2
	0.47	0.02						
	0.49	0.05						
	0.51	0.04					ICP-QQQ-MS	3
	0.51	0.02	0.51	0.52		0.52	TIMS	4
	0.52	0.03						
	0.47	0.05	0.47	0.50	0.03	0.53	ICP-QQQ-MS	5
	0.53	0.08						
	0.48	0.05						
	0.52	0.17						
	0.51	0.04					TIMS	6

a. All the measured $^{135}Cs/^{137}Cs$ ratio data corrected to 11 March 2011

Table S4. Comprehensive comparison of $^{135}Cs/^{137}Cs$ ratio for IAEA reference material (IAEA-372) [a]

IAEA reference material	$^{135}Cs/^{137}Cs$		Statistics				Instrument	Ref.
	Ratio	Uncertainty	Min	Median	SD	Max		
IAEA-372	0.54	0.03	0.50	0.54	0.02	0.56	ICP-QQQ-MS	This work
	0.50	0.04						
	0.53	0.03						
	0.54	0.04						
	0.54	0.03						
	0.53	0.03						
	0.54	0.03						
	0.56	0.09						
	0.52	0.00	0.52	0.52	0.01	0.53	TIMS	1
	0.52	0.00						
	0.52	0.00						
	0.52	0.00						
	0.53	0.01						
	0.53	0.01						
	0.53	0.02						
	0.47	0.00	0.47	0.48	0.02	0.51	ICP-QQQ-MS	2
	0.50	0.03						
	0.48	0.00						
	0.47	0.01						
	0.49	0.01						
	0.51	0.00						
	0.47	0.01						

a. All the measured $^{135}Cs/^{137}Cs$ ratio data corrected to 11 March 2011

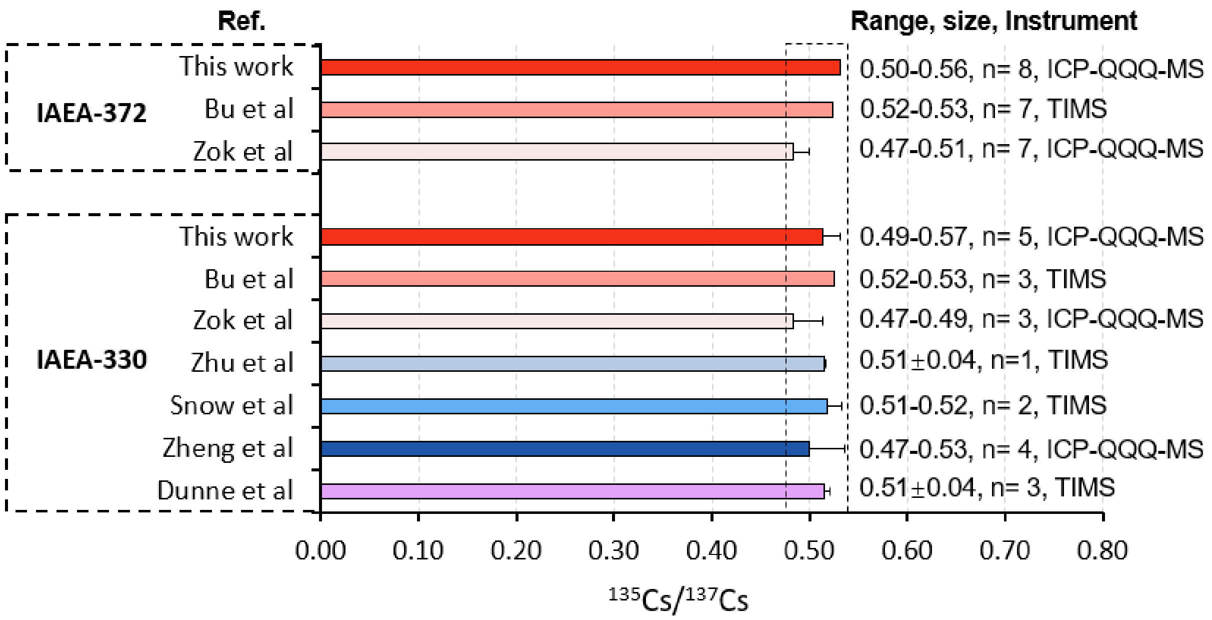

Figure S2. Comprehensive comparison of $^{135}Cs/^{137}Cs$ ratio in IAEA reference materials (IAEA-330 and IAEA-372) with the ratios reported in previous studies.

REFERENCE

1. Bu, W, et al. Ultra-trace determination of the $^{135}Cs/^{137}Cs$ isotopic ratio by thermal ionization mass spectrometry with application to Fukushima marine sediment samples. *J. Anal. At. Spectrom.* 34 (2), 301-309 (2019).
2. Zok, D, et al. Determination of characteristic vs anomalous $^{135}Cs/^{137}Cs$ isotopic ratios in radioactively contaminated environmental samples. *Environ. Sci. Technol.* 55(8), 4984-4991 (2021).
3. Zhu, L., Hou, X., & Qiao, J. Determination of ultralow level ^{135}Cs and $^{135}Cs/^{137}Cs$ ratio in environmental samples by chemical separation and triple

quadrupole ICP-MS. *Anal. Chem.* 92(11), 7884-7892 (2020).

4. Snow, M. S., & Darin C. S. $^{135}Cs/^{137}Cs$ isotopic composition of environmental samples across Europe: environmental transport and source term emission applications. *J. Environ. Radioact.* 151, 258-263 (2016).
5. Zheng, J., et al. Triple-quadrupole inductively coupled plasma-mass spectrometry with a high-efficiency sample introduction system for ultratrace determination of ^{135}Cs and ^{137}Cs in environmental samples at femtogram levels. *Anal. Chem.* 88(17), 8772-8779 (2016).
6. Dunne, A. J., Richards, A. D., & Chen. H. Procedures for precise measurements of $^{135}Cs/^{137}Cs$ atom ratios in environmental samples at extreme dynamic ranges and ultra-trace levels by thermal ionization mass spectrometry. *Talanta* 174, 347-356 (2017).

5. Detailed information of samples

Table S5. Detailed information of sampling in the study area

No.	ID	Sampling district	Longitude (°E)	Latitude (°N)	Altitude (m) [a]	^{137}Cs inventory (Bq·m^{-2}) [b]		Sampling date	Gender [c]	Animal weight (kg) [c]	Age (y) [c]
						Mean	Min. ~ Max.				
1	A	Kronach	11.32	50.45	503.65	6123	3643 ~ 8717	4/7/2020	Female	20	1.7
2	A	Kronach	11.41	50.42	503.65	6123	3643 ~ 8717	4/9/2020	Male	19	/
3	A	Kronach	11.41	50.42	503.65	6123	3643 ~ 8717	4/9/2020	Male	21	/
4	A	Kronach	11.41	50.42	503.65	6123	3643 ~ 8717	3/13/2021	Female	54	< 2.0
5	A	Kronach	11.26	50.46	503.65	6123	3643 ~ 8717	4/16/2021	Male	15	< 1.0
6	A	Kronach	11.37	50.51	503.65	6123	3643 ~ 8717	4/20/2021	Male	28	/
7	A	Kronach	11.37	50.51	503.65	6123	3643 ~ 8717	3/23/2021	Female	24	< 1.0
8	A	Kronach	11.37	50.51	503.65	6123	3643 ~ 8717	5/8/2021	Female	34	< 2.0
9	B	Schwandorf	12.27	49.26	463.50	9027	4436 ~ 14658	11/27/2019	Male	37	2.0
10	B	Schwandorf	12.27	49.18	463.50	9027	4436 ~ 14658	3/2/2020	/	27	< 1.0
11	B	Schwandorf	12.27	49.18	463.50	9027	4436 ~ 14658	3/4/2020	/	33	/

Table S5. Continued

12	C	Cham	13.04	49.17	529.55	10260	6231 ~ 43243	11/13/2019	Female	51	1.5
13	C	Cham	12.99	49.16	529.55	10260	6231 ~ 43243	11/23/2019	Female	30	2.0
14	C	Cham	13.06	49.15	529.55	10260	6231 ~ 43243	12/19/2019	Male	/	< 1.0
15	D	Regensburg	12.14	49.16	422.47	11880	6574 ~ 15873	4/12/2020	Female	40	/
16	D	Regensburg	12.14	49.16	422.47	11880	6574 ~ 15873	5/5/2020	Male	43	/
17	E	Kelheim	11.83	48.73	429.27	12716	8341 ~ 18976	11/10/2019	Male	25	< 1.0
18	E	Kelheim	11.83	48.73	429.27	12716	8341 ~ 18976	11/15/2019	Female	/	< 1.0
19	E	Kelheim	11.85	48.75	429.27	12716	8341 ~ 18976	1/8/2020	/	42	< 1.0
20	E	Kelheim	11.77	48.80	429.27	12716	8341 ~ 18976	5/3/2020	Female	62	2.0
21	F	Freising	11.74	48.40	468.28	15455	10693 ~ 19688	3/4/2020	/	/	/
22	F	Freising	11.74	48.40	468.28	15455	10693 ~ 19688	3/4/2020	/	/	/
23	G	Freyung-Grafenau	13.58	48.89	730.14	9628	6797 ~ 21293	1/11/2020	Male	70	5.0
24	H	Passau	13.39	48.40	439.54	11456	6256 ~ 19131	3/4/2020	/	36	< 2.0
25	I	Aichach	11.13	48.45	490.52	29238	19464 ~ 42493	1/8/2020	/	/	/
26	I	Aichach	11.13	48.45	490.52	29238	19464 ~ 42493	1/8/2020	Female	48	3.0

Table S5. Continued

27	I	Aichach	11.13	48.45	490.52	29238	19464 ~ 42493	1/10/2020	Female	40	3.0
28	I	Aichach	11.13	48.45	490.52	29238	19464 ~ 42493	1/18/2020	/	/	/
29	I	Aichach	11.13	48.45	490.52	29238	19464 ~ 42493	2/16/2020	Female	20	1.0
30	I	Aichach	11.13	48.45	490.52	29238	19464 ~ 42493	3/4/2020	/	/	/
31	I	Aichach	11.13	48.45	490.52	29238	19464 ~ 42493	3/4/2020	/	/	/
32	J	Landsberg	10.98	48.15	626.95	26806	19235 ~ 36655	12/23/2019	Male	38	1.5
33	J	Landsberg	10.98	48.11	626.95	26806	19235 ~ 36655	12/27/2019	Female	51	1.5
34	J	Landsberg	10.98	48.11	626.95	26806	19235 ~ 36655	12/27/2019	Female	35	1.0
35	J	Landsberg	10.98	48.11	626.95	26806	19235 ~ 36655	12/27/2019	Female	32	1.0
36	J	Landsberg	10.98	48.15	626.95	26806	19235 ~ 36655	12/30/2019	Female	43	1.5
37	K	Garmisch-Partenkirche	11.25	47.66	1116.34	31377	21407~ 45076	11/15/2019	Female	40	1.5
38	K	Garmisch-Partenkirche	11.23	47.63	1116.34	31377	21407~ 45076	11/15/2019	Female	41	1.5
39	K	Garmisch-Partenkirche	11.23	47.63	1116.34	31377	21407~ 45076	11/15/2019	Female	38	1.5
40	K	Garmisch-Partenkirche	11.30	47.68	1116.34	31377	21407~ 45076	11/29/2019	Female	40	2.0
41	K	Garmisch-Partenkirche	11.30	47.68	1116.34	31377	21407~ 45076	11/29/2019	Female	10	0.5

Table S5. Continued

42	K	Garmisch-Partenkirche	11.03	47.57	1116.34	31377	21407~ 45076	12/2/2019	Female	29	1.5
43	K	Garmisch-Partenkirche	11.09	47.71	1116.34	31377	21407~ 45076	12/8/2019	Female	31	1.0
44	K	Garmisch-Partenkirche	11.30	47.68	1116.34	31377	21407~ 45076	1/5/2020	Female	60	3.0
45	K	Garmisch-Partenkirche	10.99	47.69	1116.34	31377	21407~ 45076	1/9/2020	/	61	/
46	K	Garmisch-Partenkirche	11.03	47.57	1116.34	31377	21407~ 45076	1/9/2020	Female	9	1.0
47	K	Garmisch-Partenkirche	11.03	47.57	1116.34	31377	21407~ 45076	1/9/2020	Female	11	1.0
48	K	Garmisch-Partenkirche	11.03	47.57	1116.34	31377	21407~ 45076	1/9/2020	Male	14	1.0

a. Average altitude of district

b. The ^{137}Cs inventory data is derived from the German Federal Office for Radiation Protection. The sampling resolution in study area is about 8 × 8 km and the ^{137}Cs inventory was corrected to 1986.

c. “/” means unknown

Table S6. Detailed information of samples

No.	ID	Sampling district	Fresh weight (g) [a]	Ashed weight (g) [b]	Used weight (g)[b]	^{137}Cs activity concentration ± uncertainty (Bq·kg^{-1}) [c]	$^{137}Cs/^{133}Cs$ ± uncertainty (×10^{-21}) [d]	$^{135}Cs/^{137}Cs$ ± uncertainty [e]
1	A	Kronach	469.1	5.13885	1.3635	846 ± 19	27.1 ± 0.5	1.52 ± 0.08
2	A	Kronach	508.5	6.63595	1.25067	704 ± 16	36.3 ± 0.7	1.18 ± 0.03
3	A	Kronach	512	6.14948	2.36496	500 ± 11	54.5 ± 1.0	1.18 ± 0.05
4	A	Kronach	543.8	5.98643	1.11998	840 ± 19	30.9 ± 0.6	1.47 ± 0.03
5	A	Kronach	373.2	4.44861	1.73267	589 ± 13	18.6 ± 0.3	1.97 ± 0.04
6	A	Kronach	444.8	4.70155	0.99278	918 ± 21	29.7 ± 0.6	1.52 ± 0.02
7	A	Kronach	428.5	4.66592	1.44477	645 ± 15	22.4 ± 0.4	1.52 ± 0.04
8	A	Kronach	481.6	4.67213	1.61017	513 ± 11	24.6 ± 0.5	1.33 ± 0.06
9	B	Schwandorf	449.1	5.24639	0.43878	1711 ± 41	172.9 ± 4.1	0.79 ± 0.01
10	B	Schwandorf	583	5.69191	0.68997	2601 ± 60	101.0 ± 2.3	0.69 ± 0.02
11	B	Schwandorf	452.9	4.79285	0.47513	3150 ± 76	97.2 ± 2.4	0.67 ± 0.02
12	C	Cham	192.5	2.43372	0.53751	2180 ± 49	37.8 ± 0.7	1.1 ± 0.03
13	C	Cham	115.3	1.3924	0.16218	5705 ± 136	205.2 ± 4.2	0.71 ± 0.01

Table S6. Continued

14	C	Cham	159.5	1.80137	0.11656	9230 ± 285	117.3 ± 3.1	0.77 ± 0.02
15	D	Regensburg	543.7	5.26365	0.45295	2536 ± 60	106.6 ± 2.5	0.68 ± 0.01
16	D	Regensburg	620.8	6.67713	0.55944	2533 ± 75	92.9 ± 2.7	0.75 ± 0.02
17	E	Kelheim	425.2	5.28319	0.50535	2689 ± 64	8.8 ± 0.2	1.26 ± 0.14
18	E	Kelheim	443.3	4.8747	1.08531	1611 ± 36	103.8 ± 2.3	1.07 ± 0.02
19	E	Kelheim	520.5	4.26987	1.18529	1073 ± 21	152.7 ± 3.0	1.05 ± 0.01
20	E	Kelheim	495.3	5.67324	1.23124	763 ± 16	119.6 ± 2.6	1.19 ± 0.02
21	F	Freising	729	8.19125	1.86363	530 ± 12	74.4 ± 1.4	0.95 ± 0.03
22	F	Freising	792.1	9.64644	1.79281	575 ± 13	142.4 ± 2.8	0.9 ± 0.02
23	G	Freyung-Grafenau	477.6	5.29059	0.52588	1874 ± 49	53.5 ± 1.2	0.89 ± 0.04
24	H	Passau	484.3	4.98655	0.47285	1888 ± 49	136.3 ± 3.0	0.84 ± 0.03
25	I	Aichach	302.6	4.12206	0.84963	1746 ± 37	261.6 ± 5.6	0.8 ± 0.02
26	I	Aichach	347.9	7.18939	1.24718	1743 ± 37	326.8 ± 7.4	0.72 ± 0.02
27	I	Aichach	555.4	4.75397	1.06758	822 ± 17	267.3 ± 5.5	0.78 ± 0.02
28	I	Aichach	492.2	13.20665	1.64107	1514 ± 32	124.5 ± 2.7	0.89 ± 0.02

Table S6. Continued

29	I	Aichach	416.7	4.36888	0.33917	3842 ± 113	368.2 ± 10.9	0.76 ± 0.03
30	I	Aichach	514.6	5.08202	0.20282	7394 ± 392	424.7 ± 22.6	0.74 ± 0.07
31	I	Aichach	358.3	4.79361	0.1987	9329 ± 428	464.4 ± 21.3	0.79 ± 0.05
32	J	Landsberg	481.2	5.16268	0.88774	1310 ± 29	150.0 ± 3.3	0.87 ± 0.02
33	J	Landsberg	524	5.55685	0.77362	1517 ± 35	107.3 ± 2.5	0.82 ± 0.01
34	J	Landsberg	461.2	5.56589	0.70276	1918 ± 41	176.8 ± 3.8	0.77 ± 0.03
35	J	Landsberg	461.1	5.50425	0.72401	1885 ± 44	159.5 ± 3.7	0.75 ± 0.02
36	J	Landsberg	538.8	6.26586	1.33096	762 ± 13	123.9 ± 2.1	0.72 ± 0.04
37	K	Garmisch-Partenkirchen	555.4	6.27706	1.08764	682 ± 14	78.2 ± 1.4	0.96 ± 0.01
38	K	Garmisch-Partenkirchen	542.5	6.671	1.05785	677 ± 14	97.0 ± 1.7	0.96 ± 0.03
39	K	Garmisch-Partenkirchen	514.9	5.25369	0.45816	370 ± 8	93.2 ± 1.7	1.02 ± 0.03
40	K	Garmisch-Partenkirchen	572.7	5.37427	0.59084	1101 ± 22	92.7 ± 1.9	1.06 ± 0.01
41	K	Garmisch-Partenkirchen	506	5.55698	0.88718	1261 ± 28	96.7 ± 2.1	1.08 ± 0.02
42	K	Garmisch-Partenkirchen	500.7	5.44331	0.06361	8686 ± 768	350.4 ± 31.0	0.95 ± 0.05
43	K	Garmisch-Partenkirchen	496.3	6.69766	0.13717	2430 ± 61	229.9 ± 5.8	0.89 ± 0.07

Table S6. Continued

44	K	Garmisch-Partenkirchen	483.9	5.27128	0.12137	1773 ± 39	129.6 ± 2.8	1.12 ± 0.06
45	K	Garmisch-Partenkirchen	647.6	7.88781	0.19134	1310 ± 26	176.4 ± 3.6	0.84 ± 0.03
46	K	Garmisch-Partenkirchen	221.2	2.47172	1.05615	13887 ± 541	371.0 ± 14.5	0.92 ± 0.03
47	K	Garmisch-Partenkirchen	98.7	1.11246	1.13972	14449 ± 372	232.5 ± 6.0	0.79 ± 0.01
48	K	Garmisch-Partenkirchen	93.1	0.87649	0.91286	10978 ± 375	392.3 ± 13.4	0.91 ± 0.02

a. Weighing resolution of balance: 0.1 g

b. Weighing resolution of balance: 0.00001 g

c. Measured uncertainty of ^{137}Cs activity is given by gamma spectrometry (k=1).

d. Combined uncertainty of ^{137}Cs/^{135}Cs is estimated by the measured uncertainties of ^{137}Cs (gamma detector) and 135Cs (ICP-QQQ-MS).

e. Measured uncertainty of ^{135}Cs/^{137}Cs ratio is given by ICP-QQQ-MS (k=1). The measured value was corrected to 11 March 2011.

6. Historical variation of ^{137}Cs activity in Bavarian wild boars

Through hunters who are active in Bavaria, we obtained a long-term record of ^{137}Cs activity concentrations in the meat of wild boars collected from Aichach (region I) since 2001. Given that the ^{137}Cs activity concentration in wild boars in proximal regions has been reported to have a pronounced seasonal pattern[1], before implementing the time-series comparison, there is a need to know if similar characteristics are also present in Bavaria. For this reason, we grouped the wild boar samples collected in the same month first to check the dependence of ^{137}Cs activity concentration in the meat of wild boars with month. As shown in Figure S3, it can be observed that a significant decline in ^{137}Cs level in the summer and fall seasons, while a relatively higher ^{137}Cs contamination is shown in the winter and spring seasons. Considering that our samples were collected from November to May, the fluctuation of activity in wild boars during this period is relatively limited. Therefore, we selected the historical data obtained in the corresponding month and used the year as a classification indicator to observe the annual variation of ^{137}Cs in Bavarian wild boars.

In Figure S4, it can be noted no significant decline in ^{137}Cs activity concentration in the meat of Bavarian wild boars temporally, even though it has been over 20 years (physical half-live of ^{137}Cs is 30.08 years) since first record. Moreover,

we can find an even higher ^{137}Cs median in the 2020s compared to the one recorded in the 2000s. Previous studies named this unusual ^{137}Cs time series variation as the wild boar paradox[1], and similar patterns have been reported several times in the Alpine regions[2-4].

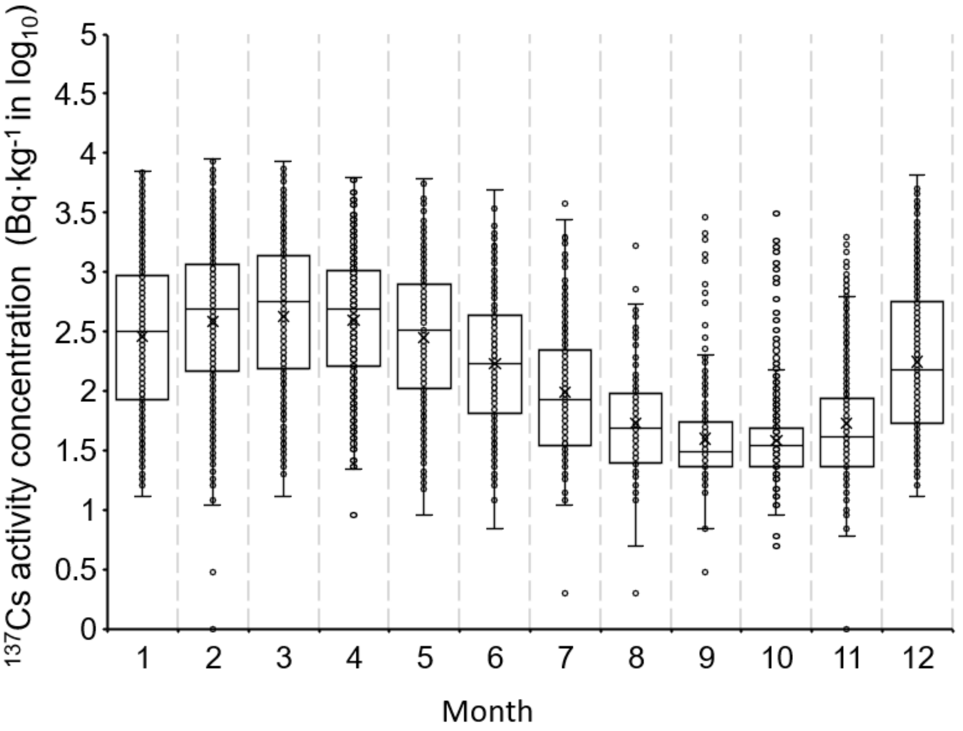

Figure S3. Seasonal variations in ^{137}Cs activity concentration (Bq·kg^{-1}, in log$_{10}$) in wild boar collected from Aichach from 2001 to 2022. In each box plot, the lines from the top to the bottom represent the maximum, 75% quantile, median, 25% quantile, and minimum, respectively. The circles and cross symbols mean the distribution of data and the mean value.

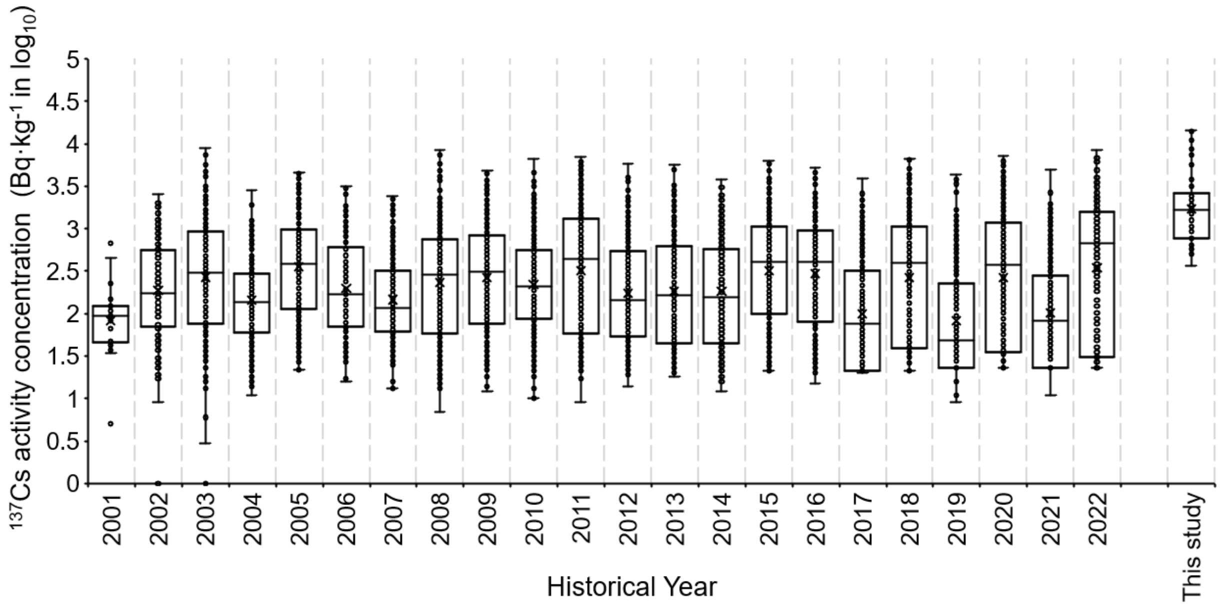

Figure S4. Comparison of annual variations in ^{137}Cs activity concentration (Bq·kg^{-1}, in $\log_{10}$) in wild boars collected from Aichach, Bavaria (November to May from 2001 to 2022) and the samples measured in this study. In each box plot, the lines from the top to the bottom represent the maximum, 75% quantile, median, 25% quantile, and minimum, respectively. The circles and cross symbols mean the distribution of data and the mean value.

REFERENCES:

1. Berendes, O., & Steinhauser, G. Exemplifying the "wild boar paradox": dynamics of cesium-137 contaminations in wild boars in Germany and Japan. *J. Radioanal. Nucl. Chem.* **2022**, 331, 5003–5012.
2. Strebl, F. & Tataruch, F. Time trends (1986–2003) of radiocesium transfer to roe deer and wild boar in two Austrian forest regions. *J. Environ. Radioact.*

2007, 98, 137-152.

3. Semizhon, T., Putyrskaya, V., Zibold, G. & Klemt, E. Time-dependency of the ^{137}Cs contamination of wild boar from a region in Southern Germany in the years 1998 to 2008. *J. Environ. Radioact.* **2009**, 100, 988-992.
4. Steinhauser, G., & Saey, P.R.J. ^{137}Cs in the meat of wild boars: a comparison of the impacts of Chernobyl and Fukushima. *J. Radioanal. Nucl. Chem.* **2016**, 307, 1801-1806.

7. Comparison of measured $^{135}Cs/^{137}Cs$ ratios with reported values

With the rapid development of analytical methods for measuring $^{135}Cs/^{137}Cs$ via using commercially available instruments, such as thermal ionization mass spectrometry (TIMS) and triple quadrupole ICP-mass spectrometry (ICP-QQQ-MS) in recent two decades, an increasing number of studies have successfully achieved the determination of $^{135}Cs/^{137}Cs$ ratios in the samples collected from various environments. Depending on the sampling sites, it is well known that the reported $^{135}Cs/^{137}Cs$ ratios can exhibit significant spatial variations. To better summarize the spatial patterns of $^{135}Cs/^{137}Cs$ ratios worldwide, we conducted a literature review (Web of Science) based on the keywords of "$^{135}Cs/^{137}Cs$ ratio" and "environmental samples". After that, we included the reported data in our database if the publication reported detailed information about the sampling location and measurement time. Considering the samples collected from nuclear facilities or decommissioning sites may have a more complicated radiocesium mixing process, we did not involve them in our database, as this work mainly focused on the $^{135}Cs/^{137}Cs$ ratios in samples collected from general environments. As a result, a total of 139 records were gathered and summarized in Tables S7-9 [1-12], which were used for comparison with our data. Similarly, all reported ratios were corrected to 11 March 2011 for convenient comparison.

Table 7. Reported $^{135}Cs/^{137}Cs$ ratio in the environmental samples collected from Ukraine

Regions	$^{135}Cs/^{137}Cs$		Statistics		Sample types	Instrument	Ref.
	Ratio	Uncertainty	Median	SD			
Ukraine	0.50	0.00	0.50	0.03	catfish	ICP-QQQ-MS	1
	0.49	0.01			catfish		
	0.49	0.00			catfish		
	0.49	0.00			catfish		
	0.51	0.01			catfish		
	0.48	0.00			moss		
	0.49	0.05			moss		
	0.48	0.00			moss		
	0.50	0.01			moss		
	0.49	0.00			moss		
	0.50	0.01			zander		
	0.50	0.00			zander		
	0.52	0.01			zander		
	0.53	0.06			soil	ICP-QQQ-MS	2
	0.58	0.07			soil		
	0.58	0.07			soil		

Table 8. Reported $^{135}Cs/^{137}Cs$ ratio in the environmental samples collected from Japan

Regions	$^{135}Cs/^{137}Cs$		Statistics		Sample types	Instrument	Ref.
	Ratio	Uncertainty	Median	SD			
Japan	0.39	0.14	0.36	0.07	rainfall	ICP-QQQ-MS	3
	0.31	0.10			rainfall		
	0.37	0.01			rainfall		
	0.35	0.02			rainfall		
	0.36	0.03			rainfall		
	0.35	0.02			rainfall		
	0.40	0.04			sediment	ICP-QQQ-MS	4
	0.36	0.02			sediment		
	0.39	0.06			sediment		
	0.39	0.05			sediment		
	0.43	0.07			sediment		
	0.45	0.09			sediment		
	0.41	0.05			sediment		
	0.33	0.01			litter		
	0.34	0.00			litter		
	0.34	0.00			litter	ICP-QQQ-MS	5
	0.34	0.02			lichen		
	0.34	0.01			litter		
	0.38	0.02			soil		
	0.35	0.00			moss	ICP-QQQ-MS	1
	0.38	0.02			moss		
	0.35	0.00			moss		
	0.34	0.00			moss		
	0.35	0.00			moss		
	0.35	0.00			moss		
	0.35	0.00			moss		
	0.37	0.01			moss		
	0.38	0.00			grass	TIMS	6
	0.39	0.00			bark		
	0.38	0.00			grass		

Table S8. Continued

	0.39	0.00			moss	TIMS	6
	0.33	0.01			litter	ICP-QQQ-MS	7
	0.34	0.00			litter		
	0.34	0.00			litter		
	0.34	0.02			lichen		
	0.34	0.00			litter		
	0.38	0.02			soil		
	0.34	0.02			litter		
	0.36	0.02			soil		
	0.36	0.00			litter	TIMS	8
	0.36	0.00			seawater		
Japan	0.37	0.00	0.36	0.07	soil	TIMS	9
	0.36	0.00			soil		
	0.37	0.00			soil		
	0.37	0.00			soil		
	0.36	0.00			soil		
	0.36	0.00			soil		
	0.38	0.01			soil		
	0.36	0.01			soil		
	0.67	0.05			soil		
	0.73	0.08			soil		
	0.35	0.01			soil		
	0.40	0.03			soil		
	0.40	0.03			Soil		
	0.37	0.01			Soil		
	0.50	0.01			Soil		

Table S9. Reported $^{135}Cs/^{137}Cs$ ratio in the environmental samples collected from the regions without nuclear accident

Regions	$^{135}Cs/^{137}Cs$		Statistics		Sample types	Instrument	Ref.
	Ratio	Uncertainty	Median	SD			
USA	2.84	0.53	2.10	0.40	sediment	TIMS	10
	2.10	0.53			sediment		
	2.21	0.63			sediment		
Canada	1.41	0.06	1.57	0.30	sediment	DRC-ICP-MS	11
	1.86	0.46			sediment		
	1.21	0.29			sediment		
	1.73	0.20			sediment		
Demark	0.81	0.07	0.93	0.51	seawater	TIMS	12
	0.82	0.12			seawater		
	0.97	0.10			seawater		
	0.91	0.17			seawater		
	0.80	0.22			seawater		
	0.96	0.11			seawater		
	2.18	0.43			soil	ICP-QQQ-MS	2
	1.69	0.25			soil		
Greenland	2.45	0.71			seawater	TIMS	12
	1.89	0.70			seawater		
Sweden	0.54	0.06			soil	ICP-QQQ-MS	2

REFERENCES:

1. Zok, D. et al. Determination of characteristic vs anomalous $^{135}Cs/^{137}Cs$ isotopic ratios in radioactively contaminated environmental samples. *Environ. Sci. Technol.* **2012**, 55, 1984-4991.

2. Zhu, L., Hou, X., & Qiao, J. Determination of ultralow level ^{135}Cs and $^{135}Cs/^{137}Cs$ ratio in environmental samples by chemical separation and triple quadrupole ICP-MS. *Anal. Chem.* **2020**, 92, 7884-7892.
3. Ohno, T., & Muramatsu Y. Determination of radioactive cesium isotope ratios by triple quadrupole ICP-MS and its application to rainwater following the Fukushima Daiichi Nuclear Power Plant accident. *J. Anal. At. Spectrom.* **2014**, 29, 347-351.
4. Bu, W. et al. Ultra-trace determination of $^{135}Cs/^{137}Cs$ isotopic ratio by thermal ionization mass spectrometry with application to Fukushima marine sediment samples. *J. Anal. At. Spectrom.* **2019**, 34, 301-309.
5. Zheng, J. et al. Isotopic ratio of $^{135}Cs/^{137}Cs$ as a new tracer of radiocesium released from the Fukushima nuclear accident. *Environ. Sci. Technol.* **2014**, 48, 5433-5438.
6. Shibahara, Y., et al. Analysis of cesium isotope compositions in environmental samples by thermal ionization mass spectrometry–1. A preliminary study for source analysis of radioactive contamination in Fukushima prefecture. *J Nucl Sci Technol.* **2014**, 51, 575-579.
7. Zheng, J. et al. Determination of ^{135}Cs and $^{135}Cs/^{137}Cs$ atomic ratio in environmental samples by combining ammonium molybdophosphate (AMP)-selective Cs adsorption and ion-exchange chromatographic separation to

triple-quadrupole inductively coupled plasma–mass spectrometry. *Anal. Chem.* **2014**, 86, 7103-7110.

8. Takahashi, T. Radiological Issues for Fukushima's Revitalized Future Ch. 3 (Springer Nature, 2015).
9. Shibahara, Y. et al. Analysis of cesium isotope compositions in environmental samples by thermal ionization mass spectrometry-3: measurement of isotopic ratios of cs in soil samples obtained in Fukushima prefecture. *J. Nucl. Sci. Technol.* **2017**, 54, 158-166.
10. Snyder, D. C. et al. Radioactive cesium isotope ratios as a tool for determining dispersal and re-dispersal mechanisms downwind from the Nevada Nuclear Security Site. *J. Environ. Radioact.* **2012**, 110, 46-52.
11. Taylor, V. F., Evans, R. D. & Cornett, R. J. Preliminary evaluation of $^{135}Cs/^{137}Cs$ as a forensic tool for identifying source of radioactive contamination. *J. Environ. Radioact.* **2008**, 99, 109-118.
12. Zhu, L. et al. Determination of ultratrace level ^{135}Cs and $^{135}Cs/^{137}Cs$ ratio in small volume seawater by chemical separation and thermal ionization mass spectrometry. *Anal. Chem.* **2020**, 92, 6709-6718.

8. Spatial distribution of $^{135}Cs/^{137}Cs$ ratio in Bavarian wild boars

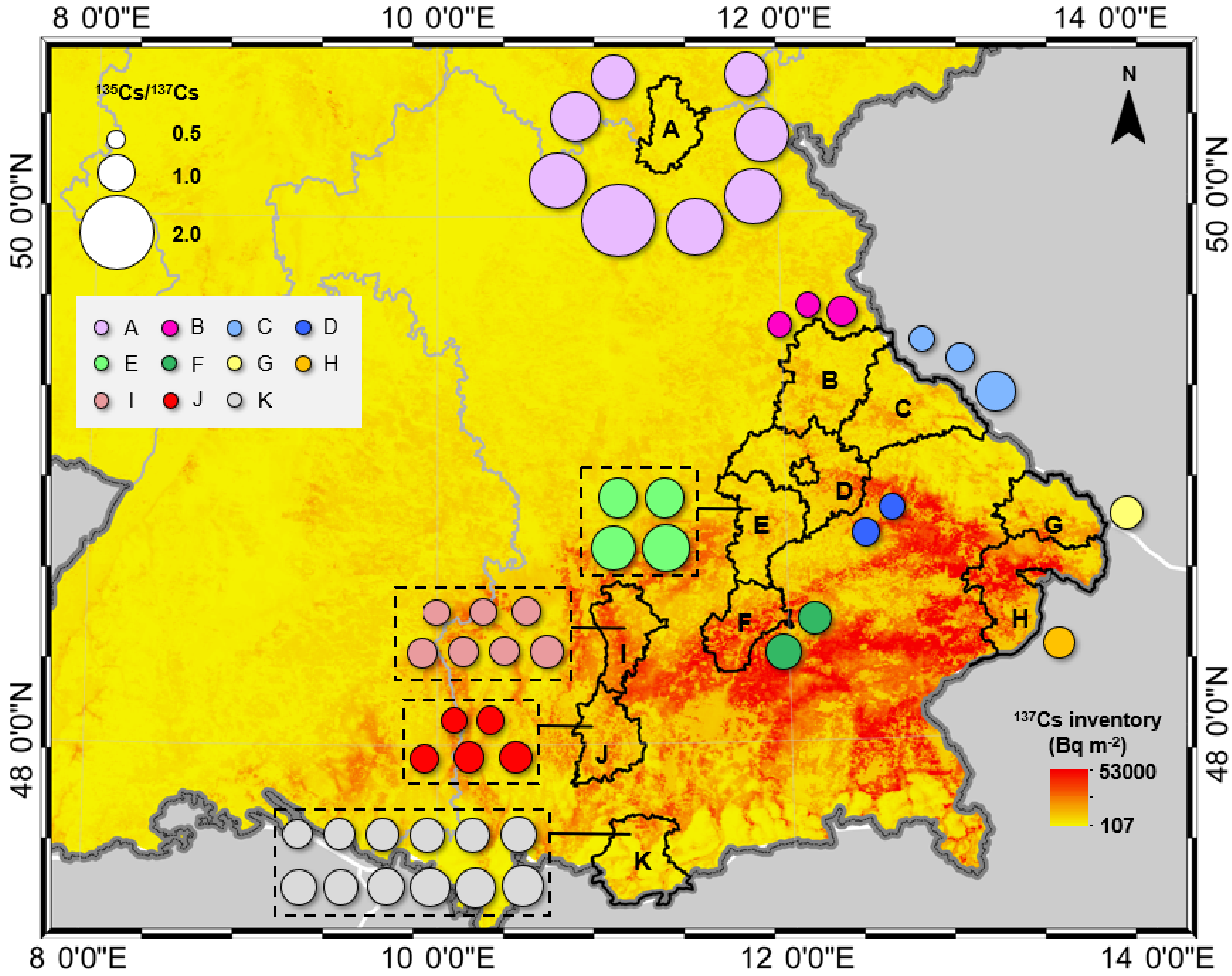

Figure S5. $^{135}Cs/^{137}Cs$ ratio in Bavarian wild boars. The ratio values were corrected to March 11, 2011. The ^{137}Cs inventory map ($Bq \cdot m^{-2}$) is derived from BfS (reference year: 1986).

9. Effects of wild boar characteristics on ^{137}Cs activity concentration and ^{135}Cs/^{137}Cs ratio

We hypothesized that the individual characteristics of the wild boars, such as gender, age, and weight, might lead to metabolic differences of ^{137}Cs in their body, whereas the ^{135}Cs/^{137}Cs ratios was not likely to be affected by these parameters, thus resulting in a mismatch between ^{137}Cs contamination levels and ^{135}Cs/^{137}Cs ratios in our wild boar samples. To validate this idea, we regrouped wild boars according to their gender (female, male, and ND), age (⩽1, 1~2, ⩾2, and ND), and weight (⩽20, 20~40, ⩾40, and ND) and used the analysis of variance for testing if the individual parameters significantly affected the ^{137}Cs levels and ^{135}Cs/^{137}Cs ratios in Bavarian wild boars. As shown in Figure S6, no significant differences ($P > 0.05$) can be found in any groups for both ^{137}Cs activity concentration and ^{135}Cs/^{137}Cs ratio. Therefore, without overemphasizing our finding, we suggest that the individual metabolic difference may be not the key factor for this "mismatch phenomenon." In the future, there is still a need to collect more comprehensive information in wild boars for quantitatively assessing the impacts caused by such individual differences on their ^{137}Cs contamination, which would provide more convincing evidence to test this hypothesis.

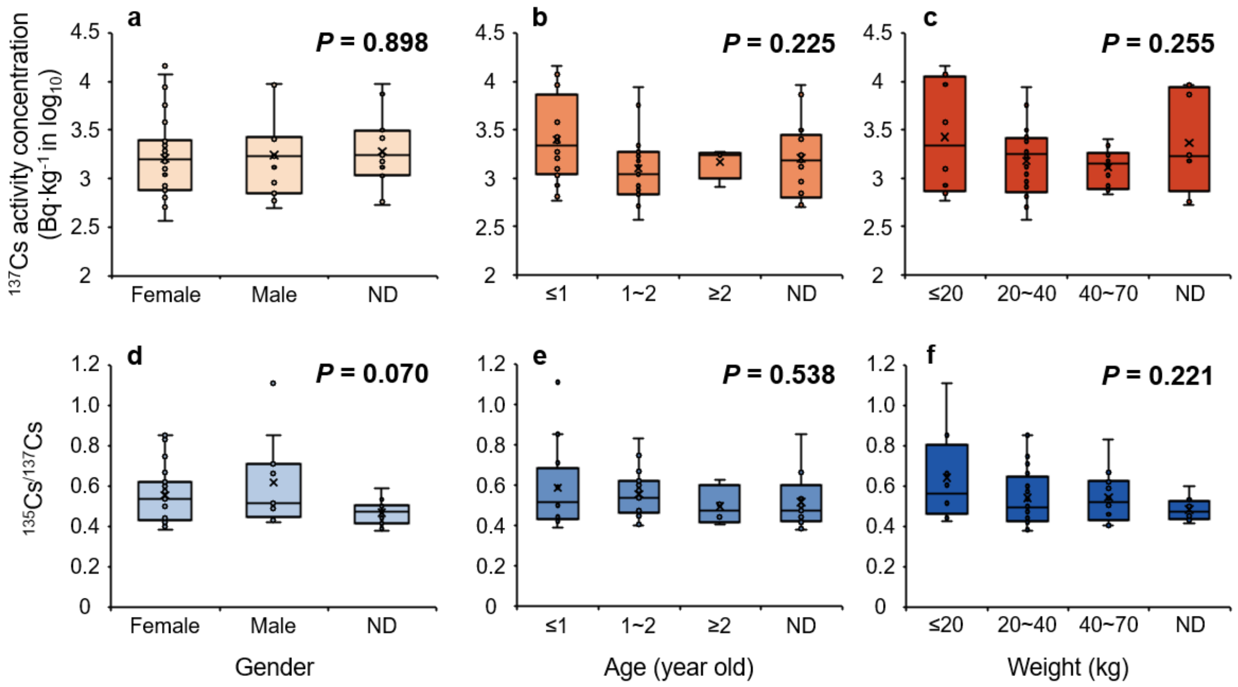

Figure S6. Difference of ^{137}Cs activity concentration (Bq·kg^{-1} in log$_{10}$) in wild boar among different gender **(a)**, age **(b)** and weight **(c)** in this study. Difference of ^{135}Cs/^{137}Cs ratio in wild boar among different gender **(d)**, age **(e)** and weight **(f)** in this study. ND means no data.

10. Relationship between ^{137}Cs activity concentration and $^{135}Cs/^{137}Cs$ ratio in each district

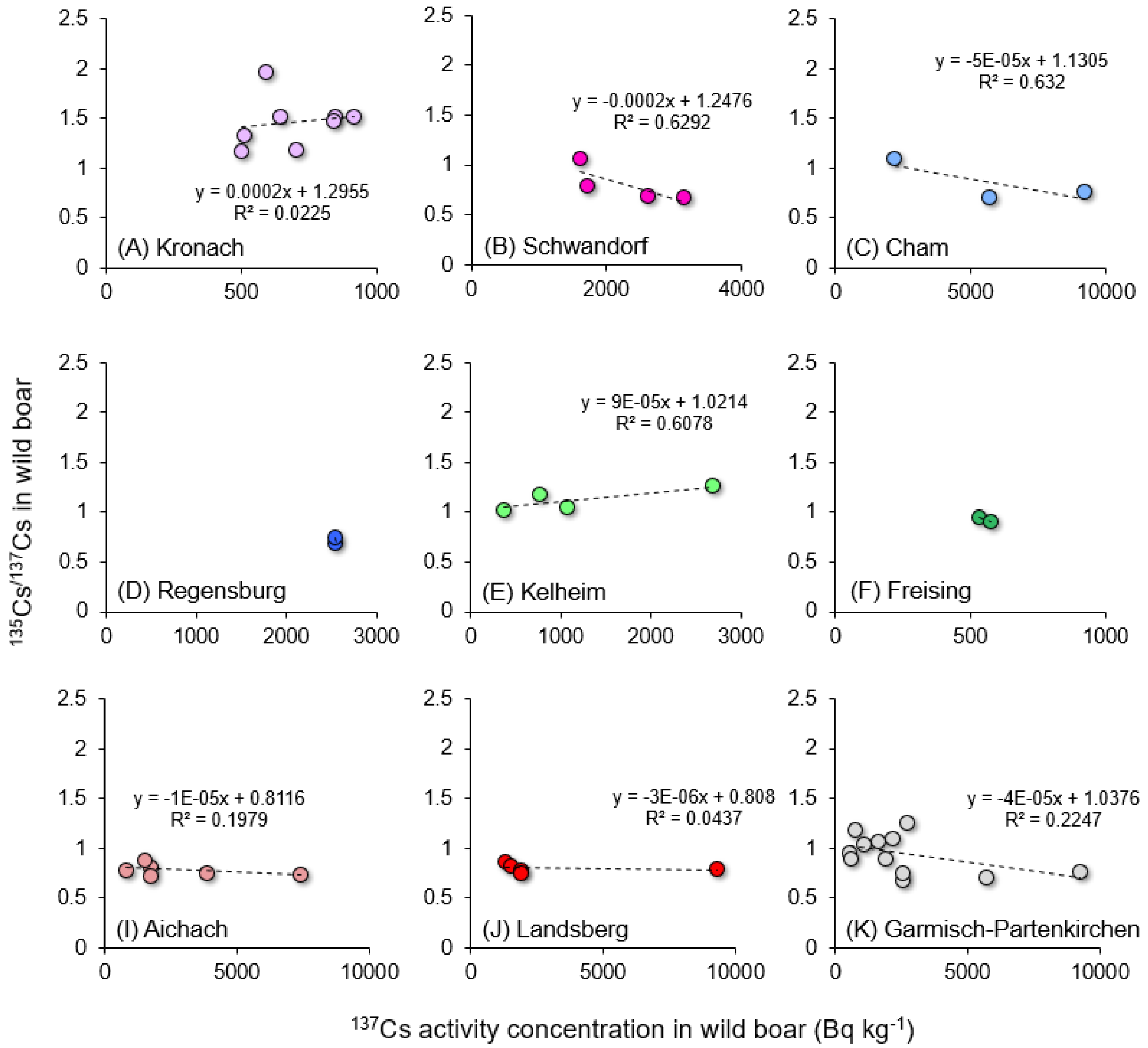

Figure S7. Relationship between the measured ^{137}Cs activity concentrations and $^{135}Cs/^{137}Cs$ ratios in Bavarian wild boar in each district (sample size ⩾ 2).

11. Estimation of ^{137}Cs contribution by mixing model

To estimate the contributions of ^{137}Cs from the Chornobyl nuclear accident and the weapons fallout in wild boar, a simple binary mixing model was performed in this study. The $^{135}Cs/^{137}Cs$ ratios in the historical human lung tissue collected from Vienna in the 1960s[1,2] (R_f= 1.92 ± 0.19, n = 5) and the IAEA reference materials (R_c = 0.53 ± 0.05, n = 13) were set as the fingerprints of weapons-^{137}Cs and Chornobyl-^{137}Cs, respectively. Physical decay correction was also performed in these measured ratios to 11 March 2011. Therefore, the percentage of weapons-^{137}Cs accounting for the ^{137}Cs contamination in a wild boar sample was calculated by following equation:

$$P_s = \frac{(R_s - R_c)}{(R_f - R_c)} \quad (5)$$

where R_s is the $^{135}Cs/^{137}Cs$ ratio measured in wild boar sample.

The uncertainty of P_s (U, k=1) was evaluated by the error propagation of each component.

$$\mathrm{U}(P) = \sqrt{\left(\frac{\partial P}{\partial R_s} \times \sigma(u_{Rs})\right)^2 + \left(\frac{\partial P}{\partial R_c} \times \sigma(u_{Rc})\right)^2 + \left(\frac{\partial P}{\partial R_f} \times \sigma(u_{Rf})\right)^2} \quad (6)$$

where $\sigma(u_{Rs})$, $\sigma(u_{Rc})$ and $\sigma(u_{Rf})$ represent the uncertainty of measured $^{135}Cs/^{137}Cs$ ratio in Bavarian wild boar samples, standard deviation of moss samples from the Chornobyl Exclusion Zone, and standard deviation of historical human lung tissue samples from Vienna, respectively. The contributions of nuclear weapon fallout to total ^{137}Cs activity concentration were listed in Table S10.

Table S10. Estimation of ^{137}Cs from the nuclear weapon fallout to total ^{137}Cs activity concentration in the meat of Bavarian wild boars.

ID	Sampling District	^{137}Cs activity concentration ± uncertainty(Bq·kg^{-1}) [a]	Fallout contribution ± uncertainty(%) [b]	Fallout ^{137}Cs activity concentration ± uncertainty (Bq·kg^{-1}) [c]	Note on regulatory limit (600 Bq·kg^{-1})
1	A	846 ± 19	67 ± 10	571 ± 87	
2	A	704 ± 16	44 ± 6	312 ± 47	
3	A	500 ± 11	44 ± 7	221 ± 35	
4	A	840 ± 19	65 ± 9	542 ± 74	
5	A	589 ± 13	99 ± 13	581 ± 77	
6	A	918 ± 21	67 ± 9	619 ± 82	exceedance
7	A	645 ± 15	68 ± 9	435 ± 60	
8	A	513 ± 11	55 ± 8	280 ± 42	
9	B	1711 ± 41	18 ± 4	304 ± 64	
10	B	2601 ± 60	11 ± 4	277 ± 97	
11	B	3150 ± 76	10 ± 4	305 ± 111	
12	C	2180 ± 49	39 ± 6	848 ± 130	exceedance
13	C	5705 ± 136	12 ± 4	702 ± 201	exceedance
14	C	9230 ± 285	16 ± 4	1476 ± 358	exceedance
15	D	2536 ± 60	10 ± 4	263 ± 89	
16	D	2533 ± 75	15 ± 4	377 ± 95	
17	E	2689 ± 64	50 ± 11	1345 ± 309	exceedance
18	E	1611 ± 36	36 ± 5	588 ± 87	
19	E	1073 ± 21	35 ± 5	377 ± 55	
20	E	763 ± 16	45 ± 6	342 ± 49	
21	F	530 ± 12	28 ± 5	151 ± 26	
22	F	575 ± 13	25 ± 4	145 ± 26	
23	G	1874 ± 49	25 ± 5	464 ± 91	
24	H	1888 ± 49	21 ± 4	403 ± 81	
25	I	1746 ± 37	18 ± 4	322 ± 70	
26	I	1743 ± 37	13 ± 4	224 ± 63	
27	I	822 ± 17	17 ± 4	142 ± 32	
28	I	1514 ± 32	24 ± 4	366 ± 67	

Table S10. Continued

29	I	3842 ± 113	15 ± 4	587 ± 162	
30	I	7394 ± 392	14 ± 6	1040 ± 433	exceedance
31	I	9329 ± 428	17 ± 5	1629 ± 487	exceedance
32	J	1310 ± 29	23 ± 4	300 ± 54	
33	J	1517 ± 35	20 ± 4	297 ± 59	
34	J	1918 ± 41	17 ± 4	316 ± 77	
35	J	1885 ± 44	15 ± 4	277 ± 73	
36	J	762 ± 13	13 ± 4	98 ± 32	
37	K	682 ± 14	29 ± 5	200 ± 32	
38	K	677 ± 14	29 ± 5	197 ± 34	
39	K	370 ± 8	33 ± 5	124 ± 20	
40	K	1101 ± 22	36 ± 5	401 ± 58	
41	K	1261 ± 28	37 ± 5	470 ± 68	
42	K	8686 ± 768	29 ± 6	2496 ± 543	exceedance
43	K	2430 ± 61	24 ± 6	588 ± 154	
44	K	1773 ± 39	40 ± 7	709 ± 122	exceedance
45	K	1310 ± 26	21 ± 4	276 ± 56	
46	K	13887 ± 541	26 ± 5	3643 ± 678	exceedance
47	K	14449 ± 372	17 ± 4	2509 ± 544	exceedance
48	K	10978 ± 375	26 ± 4	2865 ± 498	exceedance

a. Measured uncertainty of ^{137}Cs activity is given by gamma spectrometry (k=1).

b. Combined uncertainty estimated by error propagation of each component (k=1).

c. Combined uncertainty estimated by error propagation of two related uncertainties.

REFERENCES:

1. Zok, D. et al. Determination of characteristic vs anomalous ^{135}Cs/^{137}Cs isotopic ratios in radioactively contaminated environmental samples. *Environ. Sci. Technol.* **2021**, 55, 1984-4991.
2. Schönfeld, T., Liebscher, K., Karl, F., and Friedmann, C. Radioactive fission products in lungs. *Nature*, **1960**, 185, 192-193.

12. Comparison of Chornobyl-^{137}Cs contribution estimated by two methods

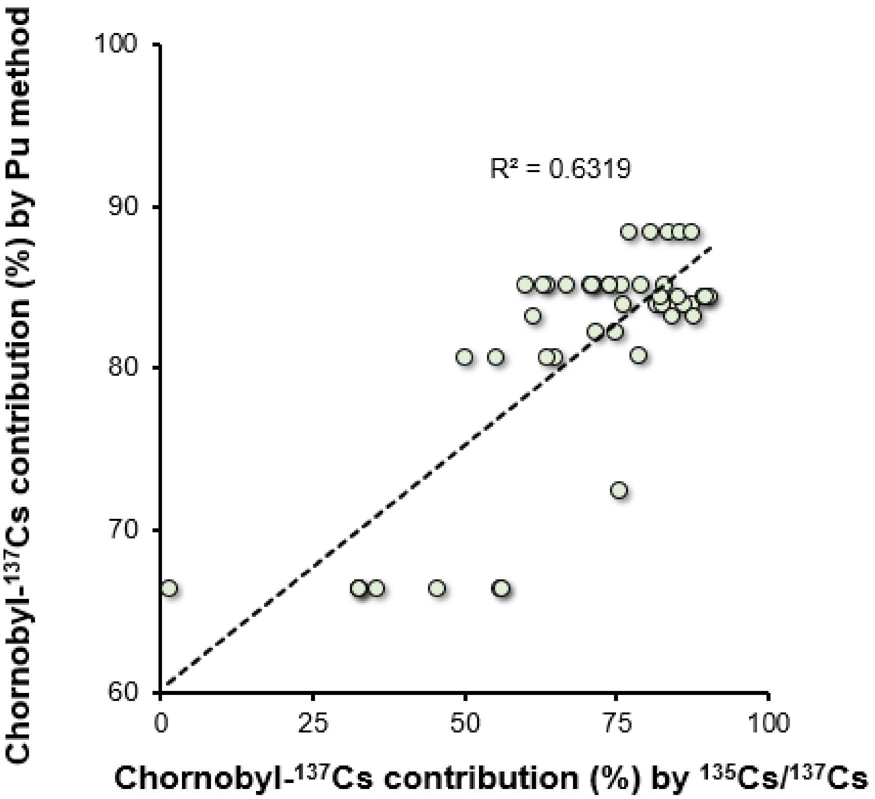

Figure S8. Comparison of the Chornobyl-^{137}Cs contribution in environmental samples from the Bavarian district: wild boar reported in this study (using ^{135}Cs/^{137}Cs) vs. surface soil reported by Meusburger et al.[1] (using Pu isotope method).

REFERENCE:

1. Meusburger, K. et al. Plutonium aided reconstruction of caesium atmospheric fallout in European topsoils. *Sci. Rep.* **2020**, 10, 11858.
